# ER-PM membrane contact site regulation by yeast ORPs and membrane stress pathways

**DOI:** 10.1371/journal.pgen.1010106

**Published:** 2022-03-03

**Authors:** Evan Quon, Aleksa Nenadic, Mohammad F. Zaman, Jesper Johansen, Christopher T. Beh

**Affiliations:** 1 Department of Molecular Biology and Biochemistry, Simon Fraser University, Burnaby, British Columbia, Canada; 2 Centre for Cell Biology, Development, and Disease, Simon Fraser University, Burnaby, Canada; The University of North Carolina at Chapel Hill, UNITED STATES

## Abstract

In yeast, at least seven proteins (Ice2p, Ist2p, Scs2/22p, Tcb1-Tcb3p) affect cortical endoplasmic reticulum (ER) tethering and contact with the plasma membrane (PM). In Δ-super-tether (Δ-s-tether) cells that lack these tethers, cortical ER-PM association is all but gone. Yeast OSBP homologue (Osh) proteins are also implicated in membrane contact site (MCS) assembly, perhaps as subunits for multicomponent tethers, though their function at MCSs involves intermembrane lipid transfer. Paradoxically, when analyzed by fluorescence and electron microscopy, the elimination of the *OSH* gene family does not reduce cortical ER-PM association but dramatically increases it. In response to the inactivation of all Osh proteins, the yeast E-Syt (extended-synaptotagmin) homologue Tcb3p is post-transcriptionally upregulated thereby generating additional Tcb3p-dependent ER-PM MCSs for recruiting more cortical ER to the PM. Although the elimination of *OSH* genes and the deletion of ER-PM tether genes have divergent effects on cortical ER-PM association, both elicit the Environmental Stress Response (ESR). Through comparisons of transcriptomic profiles of cells lacking *OSH* genes or ER-PM tethers, changes in ESR expression are partially manifested through the induction of the HOG (high-osmolarity glycerol) PM stress pathway or the ER-specific UPR (unfolded protein response) pathway, respectively. Defects in either UPR or HOG pathways also increase ER-PM MCSs, and expression of extra “artificial ER-PM membrane staples” rescues growth of UPR mutants challenged with lethal ER stress. Transcriptome analysis of *OSH* and Δ-s-tether mutants also revealed dysregulation of inositol-dependent phospholipid gene expression, and the combined lethality of *osh4*Δ and Δ-s-tether mutations is suppressed by overexpression of the phosphatidic acid biosynthetic gene, *DGK1*. These findings establish that the Tcb3p tether is induced by ER and PM stresses and ER-PM MCSs augment responses to membrane stresses, which are integrated through the broader ESR pathway.

## Author summary

Membrane contact sites (MCSs) between the two largest cellular membranes, the endoplasmic reticulum (ER) and the plasma membrane (PM), are regulatory interfaces for lipid synthesis and bidirectional transport. The yeast Osh protein family, which represents the seven yeast oxysterol-binding protein related proteins (ORPs), is implicated in MCS regulation and lipid transfer between membranes. Ironically, we find that when all Osh proteins eliminated, ER-PM association is not reduced but significantly increases. We hypothesized this increase is due to compensatory increases in levels of tether proteins that physically link the ER and PM. In fact, in response to inactivating Osh protein expression, amounts of the tether protein Tcb3 increase and more ER-PM MCSs are produced. By testing the genomic transcriptional responses to the elimination of *OSH* and ER-PM tether genes, we find these mutants disrupt phospholipid regulation and they elicit the Environmental Stress Response (ESR) pathway, which integrates many different responses needed for recovery after cellular stress. *OSH* and ER-PM tether genes affect specific stress response pathways that impact the PM and ER, respectively. Combining *OSH* and tether mutations results in cell lethality, but these cells survive by increased expression of a key phospholipid biosynthetic gene. Based on these results, we propose that *OSH* and ER-PM tether genes affect phospholipid regulation and protect the PM and ER through membrane stress responses integrated through the ESR pathway.

## Introduction

The endoplasmic reticulum (ER) is the major source of cellular lipids within the cell and most lipids are eventually targeted to the plasma membrane (PM). In addition to the lipids delivered from the ER to the PM by vesicular transport, lipid transfer can occur directly between the cortical ER and PM at membrane contact sites (MCSs). In yeast, almost half of the inner surface of the PM is covered with closely associated cortical ER [1–3]. Tether proteins establish close physical bridges between these two membranes. In yeast, seven primary tethers (Ice2p; Ist2p; Scs2p and Scs22p; Tcb1p-Tcb3p) contribute to almost all ER-PM MCS attachment [2–6]. Apart from their potential roles as lipid transfer conduits, ER-PM MCSs are a regulatory nexus that coordinates ER phospholipid synthesis with PM lipid requirements for maintaining cell membrane integrity [3,7,8].

Primary ER-PM tethers are defined as both necessary and sufficient for membrane contact under standard growth conditions [9], and they represent ER integral proteins that connect the ER and PM through MCSs, forming ~10–35 nm intermembrane gaps [1,10]. Most, but not all, yeast ER-PM tethers are well-conserved. Scs2p and Scs22p represent the yeast homologues of vesicle-associated membrane protein (VAMP)-associated protein (VAP) [11]. Scs2p serves as a scaffold protein that binds FFAT-motif (two phenylalanines in an acidic tract) proteins involved in lipid regulation [12,13]. The tricalbins Tcb1p-Tcb3p are yeast representatives of the extended synaptotagmin (E-Syt) family of tethers [5,14]. Ist2p is an ER-localized member of the TMEM16-anoctamin family, some of which are PM ion channels, while others have phospholipid scramblase activity [15]. Either through direct or indirect mechanisms, Ice2p is a yeast-specific mediator of organelle contact. During stationary phase, Ice2p attaches the ER with lipid droplets but Ice2p also confers ER-PM association during exponential growth, which promotes cortical ER inheritance along the mother PM into the daughter bud [3,4,16]. In Δ-super-tether (Δ-s-tether) cells, eliminating these tethers reduces the association of cortical ER from 48% of the PM inner surface to 1.7%, consistent with predictions of stochastic interactions between untethered ER and the PM [3].

In addition to the tether proteins, the conserved ORP (Oxysterol-binding protein related protein) family of lipid-binding proteins, including the seven yeast Osh proteins, also have established roles in MCSs formation between several different intracellular membranes [17,18]. In *Saccharomyces cerevisiae*, the ER-PM tethers and Osh proteins are necessary for maintaining normal lipid biosynthesis and distribution [3,19–21]. In Δ-s-tether cells, the additional deletion of the otherwise nonessential gene *OSH4/KES1* (referred hereafter by its systematic name, *OSH4*) results in cell lethality [3,22]. *OSH4* encodes arguably the best studied member of the yeast Oxysterol-binding protein homologues, which affect non-vesicular and vesicular transport [23–27]. The lethal genetic interaction between *osh4*Δ and Δ-s-tether mutations suggests that *OSH4* and ER-PM MCSs affect parallel regulatory pathways, likely involving phospholipid regulation. All *OSH* genes, including *OSH4*, are dispensable for yeast growth but collectively the entire *OSH* gene family is essential and any of these genes, in the absence of the other six, can provide the essential function/s of the entire family [28]. At elevated temperatures, this shared overlapping activity can be inactivated in the *osh4-1*^ts^ *osh*Δ (*osh*Δ represents deletion of all *OSH*s and *osh4-1*^ts^ is a temperature sensitive allele) conditional mutant [29]. Apart from the genetic interactions between *OSH* and ER-PM tether genes, Osh proteins regulate vesicle membrane-PM attachment via the exocyst tethering complex during post-Golgi vesicle docking at the PM [22,30,31]. Thus, Osh proteins impact membrane tethering in a variety of contexts. Longer yeast Osh proteins, such as Osh1p-3p, have N-terminal extensions that contain FFAT (two phenylalanines in an acidic tract) domains that interact with the MCS tether protein Scs2p [12,32]. In addition, the shorter Osh proteins, Osh6p and Osh7p, both localize to ER-PM contacts [33]. Many other yeast and mammalian ORPs are also located at various MCSs where they affect membrane regulation [12,18–20,34,35]. For example, phosphatidylinositol 4-phosphate (PI4P)-dependent recruitment of Osh3p to MCSs affects phosphoinositide regulation in the PM [19]. In general, these findings suggest that ORPs play structural and/or regulatory roles in MCS formation.

ER-PM MCSs might impact membrane organization similar to changes resulting from the cortical redistribution of cytoplasmic ER during ER stress [36]. The canonical ER stress response involves the accumulation of unfolded proteins within the ER lumen that activates the unfolded protein response (UPR) pathway through the conserved single-pass ER transmembrane kinase Ire1p. Changes in ER morphology such as cytoplasmic ER expansion are also integrated into the UPR pathway, but the mechanism is distinct [36,37]. UPR-independent cortical ER expansion involves inactivation of the transcriptional repressor Opi1p, or constitutive expression of the transcriptional activators Ino2/4p. Both factors bind the inositol-sensitive upstream sequences that activates gene expression for lipid production [38]. Inositol deprivation is also a potent UPR inducer [39–43]. In this manner, phospholipid biosynthesis, ER organization and membrane stress response, are integrated mechanisms.

At the PM, changes in phospholipid and carbohydrate metabolism induce the HOG (high-osmolarity glycerol) pathway, which protects cells against osmotic membrane stress [44]. In a mitogen-activated protein kinase (MAPK) cascade, osmosensors at the PM signal through a transduction of kinase phosphorylation to the MAPK Hog1p [45]. Defects in sphingolipid biosynthesis exemplify how the HOG pathway directly responds to changes in PM lipid composition. Sphingolipid dysregulation activates the HOG pathway, and HOG-dependent changes in gene expression induce phospholipid synthesis and reduces sterols to compensate [44,46].

In this study, the molecular function of Osh proteins at ER-PM MCSs was tested. To our surprise, eliminating all Osh proteins (or just the subset of longer Osh proteins) does not reduce cortical ER-PM contact but increases ER-PM association. In a compensatory response to Osh protein inactivation, we find that levels of the ER-PM tether Tcb3p increase, which increases cortical ER-PM association and Tcb3p-dependent MCSs. As shown by genomic expression profiles, deletion of the ER-PM tether genes or inactivation of the *OSH* gene family both elicit the Environmental Stress Response (ESR) through specific activation of the UPR and HOG pathways, respectively. In turn, we find that both HOG and UPR stress pathways induce ER-PM MCSs, and in the absence of UPR signaling an “artificial ER-PM membrane staple” provides resistance to lethal ER stress. High-copy genetic suppressors of Δ-s-tether *osh4-1*^*ts*^ lethality also implicate phospholipid biosynthesis in ER-PM MCSs and *OSH* regulation. Through the ESR pathway, we propose that ER-PM MCSs and Osh proteins integrate phospholipid regulation with different membrane stress responses to maintain ER and PM integrity.

## Results

### Eliminating Osh proteins increases cortical ER-PM contact, decreases nuclear ER-vacuole association, but has no net effect on nuclear ER-lipid droplet association

**Fig 1:**
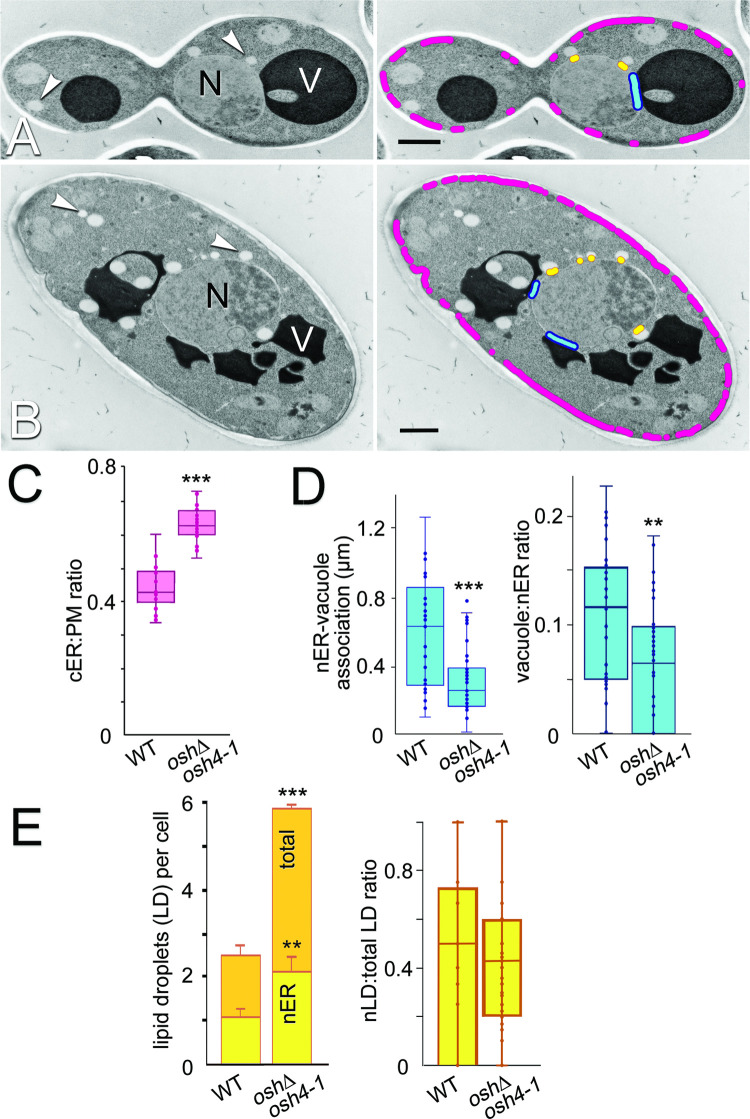
Effect of eliminating all Osh proteins on cortical ER-PM contact, nuclear ER-vacuole and nuclear ER-lipid droplet association. **(A)** Transmission electron micrographs of WT (SEY6210) and (**B)** *osh4-1*^*ts*^ *oshΔ* (CBY926) cells at 37°C for 1 h. Right panels highlight cortical ER association with the PM (magenta), nuclear ER association with vacuoles (blue), and nuclear ER association with lipid droplets (yellow). N, nucleus;V, vacuole, arrowheads indicate examples of lipid droplets. (**C)** Quantification of WT and *osh4-1*^*ts*^ *oshΔ* cell electron micrographs of the ratio of total lengths of cortical associated ER (cER) relative to the total PM lengths in WT and *osh4-1*^*ts*^ *oshΔ* cells (n = 25 cells for each strain). (**D)** Left: quantification of the average length (μm) of nuclear ER (nER) in association with vacuolar membrane in WT and *osh4-1*^*ts*^ *oshΔ* cells (n ≥ 29 cells). Right: ratio of nER associated with vacuolar membrane per the total nER (n ≥ 31 cells). (**E)** Left: comparison of total numbers of lipid droplets (LD) per cell (orange) and the number of those LDs associated with nER (yellow) in WT and *osh4-1*^*ts*^ *oshΔ* cells (n ≥ 32 cells; error bars indicate standard error of the mean [SEM]). Right: ratios of these nER-associated LDs (nLDs) to total LDs per cell (n ≥ 32 cells). In all box and whisker plots, boxes indicate the interquartile range around the mean, median values are shown by lines within boxes, error bars indicate standard deviations. All statistical significance was calculated using two tail student’s t-test with heteroscedastic variance. ***p ≤ 0.0001, **p ≤ 0.002. Scale bars = 1 μm.

ORPs, including individual yeast Osh proteins, have been implicated in the assembly of several MCSs [17,18]. To define the collective role of the Osh protein family in ER membrane associations with the PM, vacuole, and lipid storage droplets, we analyzed membrane ultrastructure in *osh4-1*^ts^ *osh*Δ cells after 1 h at 37°C by transmission electron microscopy (TEM). To our surprise, the proportion of the PM inner surface covered with cortical ER in *osh4-1*^ts^ *osh*Δ cells did not decrease when compared to wild type (WT), but rather significantly increased (Fig 1A–1C). In contrast, the elimination of all Osh proteins reduced ER-vacuole membrane association by ~2-fold, relative to WT (Fig 1A, 1B and 1D), which is consistent with the functional role of Osh1p at nuclear-vacuolar junctions [47–49]. Based on these results, the *OSH* family has opposite roles in regulating ER membrane interactions with the vacuole as opposed to the PM.

Because mammalian ORP2 plays an important role in regulating triglyceride and lipid droplet turnover [32], we determined whether nuclear ER-lipid droplet association required Osh proteins in yeast cells. Consistent with previous reports, *osh4-1*^ts^ *osh*Δ cells contain ~2-fold more lipid droplets than WT cells [29]. In *osh4-1*^ts^ *osh*Δ cells, the number of lipid droplets in contact with nuclear ER also increased ~2-fold, as a direct function of the increase in number of lipid droplets (Fig 1B and 1E). In other words, even though there are more lipid droplets in *osh4-1*^ts^ *osh*Δ cells, the proportion of lipid droplets contacting the nuclear ER compared to all lipid droplets in the cell is unchanged relative to WT (Fig 1E). Although yeast Osh proteins either affect lipid droplet biogenesis or turnover, the fraction of lipid droplets in contact with the ER is unchanged.

**Fig 2:**
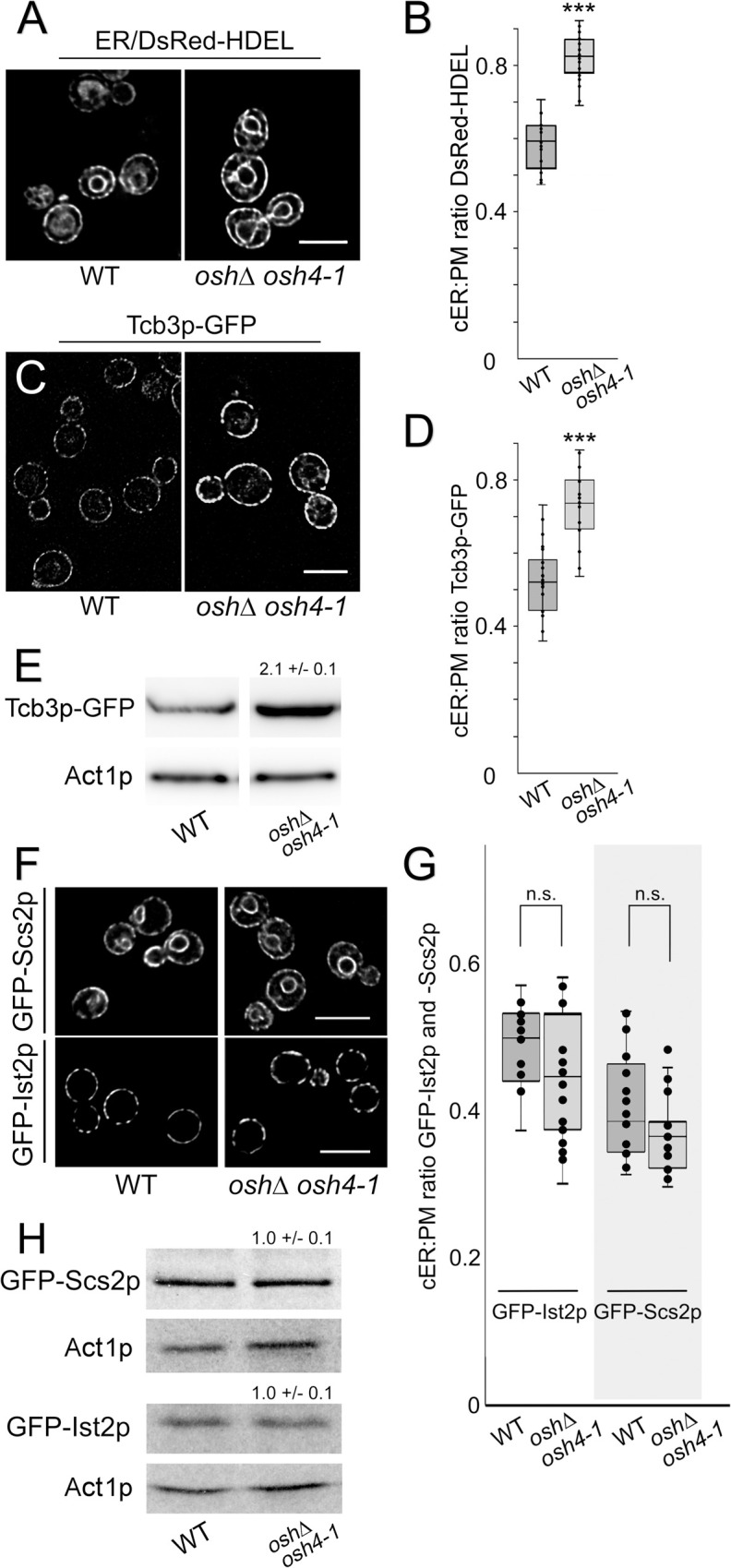
Cortical ER-PM association and levels of the Tcb3p tether increase upon inactivation of Osh proteins. **(A)** Representative fluorescent microscopy images of WT (SEY6210) and *osh4-1*^*ts*^ *oshΔ* (CBY926) cells, incubated at 37°C for 1 h, expressing episomal DsRed-HDEL (pRS416-DsRed-HDEL) showing ER localization and increased cortical fluorescence in *osh4-1*^*ts*^ *oshΔ* cells. (**B)** Quantified ratios of total lengths of DsRed-HDEL-fluorescent cER relative to total PM lengths (PM) in WT and *osh4-1*^*ts*^ *oshΔ* cells at 37°C for 1 h (n = 20 cells per strain). (**C)** Images of WT (CBY6087) and *osh4-1*^*ts*^ *oshΔ* (CBY6091) expressing integrated *TCB3*-GFP after incubation at 37°C for 1 h. (**D)** Quantified ratios of total cortical Tcb3p-GFP fluorescence length to total PM length in WT and *osh4-1*^*ts*^ *oshΔ* cells (n = 20 cells per strain). (**E)** Corresponding to panel C, representative immunoblots probed with anti-GFP and anti-actin antibodies showing relative Tcb3-GFP levels in WT and *osh4-1*^*ts*^ *oshΔ* at 37°C for 1 h, as compared to the actin (Act1p) control. In *osh4-1*^*ts*^ *oshΔ* cells the fold increase in Tcb3-GFP levels was 2.1 ± 0.1 relative to WT (mean ± SD; n = 3). (**F**) Images of WT and *osh4-1*^*ts*^ *oshΔ* cells expressing integrated GFP-*IST2* (CBY7300 and CBY7302, respectively) or integrated GFP-*SCS2* (CBY7304 and CBY7306, respectively). (**G**) Quantified ratios of total lengths of cortical GFP-Ist2p or -Scs2p fluorescence relative to total PM length in WT and *osh4-1*^*ts*^ *oshΔ* cells (n = 20 cells per strain). (**H**) Representative immunoblots probed with anti-GFP and anti-actin antibodies shown relative GFP-Ist2p or -Scs2p levels in WT and *osh4-1*^*ts*^ *oshΔ* at 37°C for 1 h, relative to the actin (Act1p) control (mean ± SD; n = 3). Box and whisker plots and statistics as described in Fig 1. ***p ≤ 9 x 10^−8^; n.s. = not significant. Scale bars = 5 μm.

To further analyze how Osh proteins impact cortical ER-PM association, DsRed-HDEL fluorescent microscopy was used to visualize the general distribution of the ER around the nucleus, within the cytoplasm, and at the cortex along the PM. Precise points of contact between the ER and PM were determined by imaging Tcb3p, which only localizes to specific ER-MCSs. As an E-Syt homologue, Tcb3p represents an established membrane tether protein that specifically attaches cortical ER to the PM. Whereas Tcb3p localization indicates active binding sites between the ER and PM, DsRed-HDEL simply shows general ER distribution and morphology as dictated by both non-specific and active mechanisms [1,10,14]. Relative to WT, after 1 h at 37°C *osh4-1*^ts^ *osh*Δ cells exhibited greater cortical ER/DsRed-HDEL association along the PM (Fig 2A and 2B). Under these conditions, GFP-Tcb3p ER-PM MCSs also increased and covered almost 75% of the PM inner surface in *osh4-1*^ts^ *osh*Δ cells, much higher than the ~50% coverage in WT cells (Fig 2C and 2D). Thus, increased cortical ER-PM association is seeded by increased GFP-Tcb3p tether expression. By immunoblot analysis, Tcb3p levels are ~2-fold higher in *osh4-1*^ts^ *osh*Δ cells as compared with WT, after a 1 h incubation at 37°C (Fig 2E). Due to Tcb3p tether upregulation, the result of eliminating Osh proteins does not reduce ER-PM MCSs but significantly increases membrane contact by attaching more ER to the PM.

To determine the specificity of Tcb3p induction, we tested if other primary ER-PM tethers are also upregulated in *osh4-1*^ts^ *osh*Δ cells to increase ER-PM association. As major contributors to ER-PM contact, we analyzed GFP-Scs2p and GFP-Ist2p localization in *osh4-1*^ts^ *osh*Δ cells (Fig 2F and 2G). Relative to WT, the punctate distribution of GFP-Ist2p at ER-PM MCSs is unchanged in *osh4-1*^ts^ *osh*Δ cells after 1 h at 37°C. GFP-Scs2p distribution and fluorescence throughout the ER in *osh4-1*^ts^ *osh*Δ cells are also the same as WT after incubating the cells at 37°C for 1 h. Immunoblot analysis confirms that both Ist2p and Scs2p expression in *osh4-1*^ts^ *osh*Δ cells is unchanged compared to WT when incubated at 37°C for 1 h (Fig 2H). In *osh4-1*^ts^ *osh*Δ cells, Tcb3p expression is specifically induced to increase ER-PM association whereas Ist2p and Scs2p are unaffected.

### Cells lacking “long” FFAT-containing Osh proteins increase cortical ER-PM association

The tether proteins Scs2p and Scs22p bind FFAT motif proteins, including the three long Osh proteins (Osh1p, Osh2p and Osh3p) [11,12]. Even though Scs2p is an ER-associated tether protein that can span the gap separating PM and cortical ER membranes, Scs2p has no intrinsic PM membrane binding domain and mutants unable to bind FFAT motifs cannot establish association with the PM [2]. It was proposed that Scs2p binds long Osh proteins as linkers to make trans contact with the PM [50,51]. If long Osh proteins mediate Scs2p tethering, then elimination of the long Osh proteins would be predicted to disrupt ER-PM MCSs like that observed in *scs2*Δ cells.

**Fig 3:**
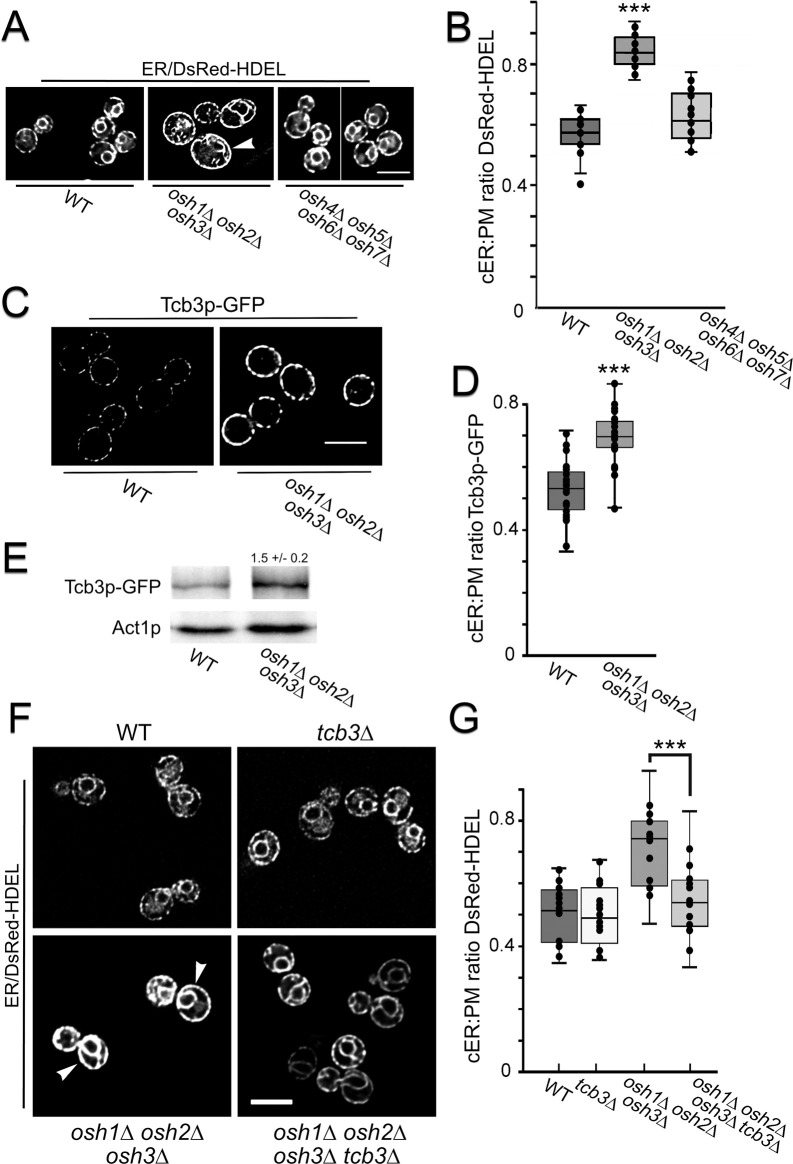
Elimination of FFAT-containing (long) Osh proteins increase Tcb3p-dependent cortical ER-PM association. **(A)** Representative fluorescent microscopy images of WT (SEY6210), *osh1*Δ *osh2*Δ *osh3*Δ (lacking “long” *OSHs*; JRY6253) and *osh4*Δ *osh5*Δ *osh6*Δ *osh7*Δ (lacking “short” *OSHs*; JRY6272) cells expressing DsRed-HDEL (pRS416-DsRed-HDEL) cultured at 30°C. (**B)** Quantified ratios for total cortical DsRed-HDEL fluorescence length per total PM length in WT, *osh1*Δ *osh2*Δ *osh3*Δ, and *osh4*Δ *osh5*Δ *osh6*Δ *osh7*Δ cells (n = 20 cells per strain). ***p = 1.9 x 10^−16^ compared to WT. (**C)** Images of WT and *osh1Δ osh2*Δ *osh3Δ* (CBY7274) expressing *TCB3*-GFP cultured at 30°C. (**D)** Quantified ratios of total cortical Tcb3p-GFP fluorescence length to total PM length in WT and *osh1Δ osh2*Δ *osh3Δ* cells (n = 20 cells per strain). ***p = 1.4 x 10^−6^ compared to WT. **(E)** Corresponding to panel C, representative immunoblots probed with anti-GFP and anti-actin antibodies showing relative Tcb3-GFP levels in WT and *osh1Δ osh2*Δ *osh3Δ*, as compared to the actin (Act1p) control. (**F**) Representative images of WT (SEY6210), *osh1*Δ *osh2*Δ *osh3*Δ (JRY6253), and *osh1*Δ *osh2*Δ *osh3*Δ *tcb3*Δ (CBY7392) cells expressing DsRed-HDEL cultured at 30°C. (**G**) Quantification of cER per total PM in WT and *osh1*Δ *osh2*Δ *osh3*Δ, and *osh1*Δ *osh2*Δ *osh3*Δ *tcb3*Δ cells corresponding to the images in F (n = 20 cells per strain). ***p = 8.2 x 10^−5^ comparing to *osh1*Δ *osh2*Δ *osh3*Δ cells with and without *TCB3* deletion. Arrowheads indicate cells with increased coverage of cortical ER. Box and whisker plots and statistics as in Fig 1. Scale bars = 5 μm.

Although elimination of all Osh proteins in *osh4-1*^ts^ *osh*Δ cells increased ER-PM contact (Fig 1A–1C), we focused on the specific impact of *osh1*Δ *osh2*Δ *osh3*Δ mutations on ER-PM association. As visualized by fluorescence microscopy, the length of cortical ER along the PM was measured in WT, *osh1*Δ *osh2*Δ *osh3*Δ, and *osh4*Δ *osh5*Δ *osh6*Δ *osh7*Δ cells (Fig 3A and 3B). In the latter mutant, the “short” Osh proteins lacking FFAT motifs are deleted [25]. In WT cells, images of the ER marker DsRed-HDEL show that ~50% of the PM is associated with cortical ER. In contrast, in *osh1*Δ *osh2*Δ *osh3*Δ cells grown at 30°C DsRed-HDEL fluorescence covers >80% of the PM. In *osh4*Δ *osh5*Δ *osh6*Δ *osh7*Δ cells, however, ER-PM association is comparable to WT. In *osh1*Δ *osh2*Δ *osh3*Δ cells, cortical GFP-Tcb3p increases to ~70%, which is comparable to the increases observed in *osh4-1*^ts^ *osh*Δ cells (Fig 3C and 3D). In addition, GFP-Tcb3p levels increase 1.5-fold in *osh1*Δ *osh2*Δ *osh3*Δ cells, though this increase is slightly less than when all *OSH* genes are eliminated (Fig 3E). The increase in ER-PM association in *osh1*Δ *osh2*Δ *osh3*Δ cells is dependent on *TCB3*. When *TCB3* is deleted in *osh1*Δ *osh2*Δ *osh3*Δ cells, DsRed-HDEL localization at the cell cortex is comparable to WT and significantly reduced compared to *osh1*Δ *osh2*Δ *osh3*Δ cells (Fig 3F and 3G). Long Osh proteins are not only dispensable for ER-PM MCSs, but in their absence cortical ER-PM association increases due to increases in Tcb3p expression; elimination of short Osh proteins has no such effect.

Despite that short *OSH* genes do not affect cortical ER-PM association, the deletion of a short *OSH* gene, namely *OSH4*, is lethal in cells lacking ER-PM tethers [3]. By themselves, neither *osh4*Δ nor *osh4-1*^ts^ mutations have a significant effect on WT yeast growth, but *OSH4* deletion in Δ-s-tether cells causes cell lethality [3,28]. However, we find that Δ-s-tether cell growth is not affected by deleting a long *OSH* gene, *OSH2* (S1 Fig). These results are consistent with the hypothesis that long Osh proteins operate with ER-PM MCS tethers but short Osh proteins act in an independent parallel functional pathway. In other words, removing long Osh proteins or ER-PM tethers affect the same process and combining their deletions does not compound the defect. These functional interactions indicate independent but complementary roles of the long versus short Osh proteins at ER-PM MCSs.

### The Environmental Stress Response (ESR) is triggered in *osh4-1*^ts^ Δ-s-tether cells

**Fig 4:**
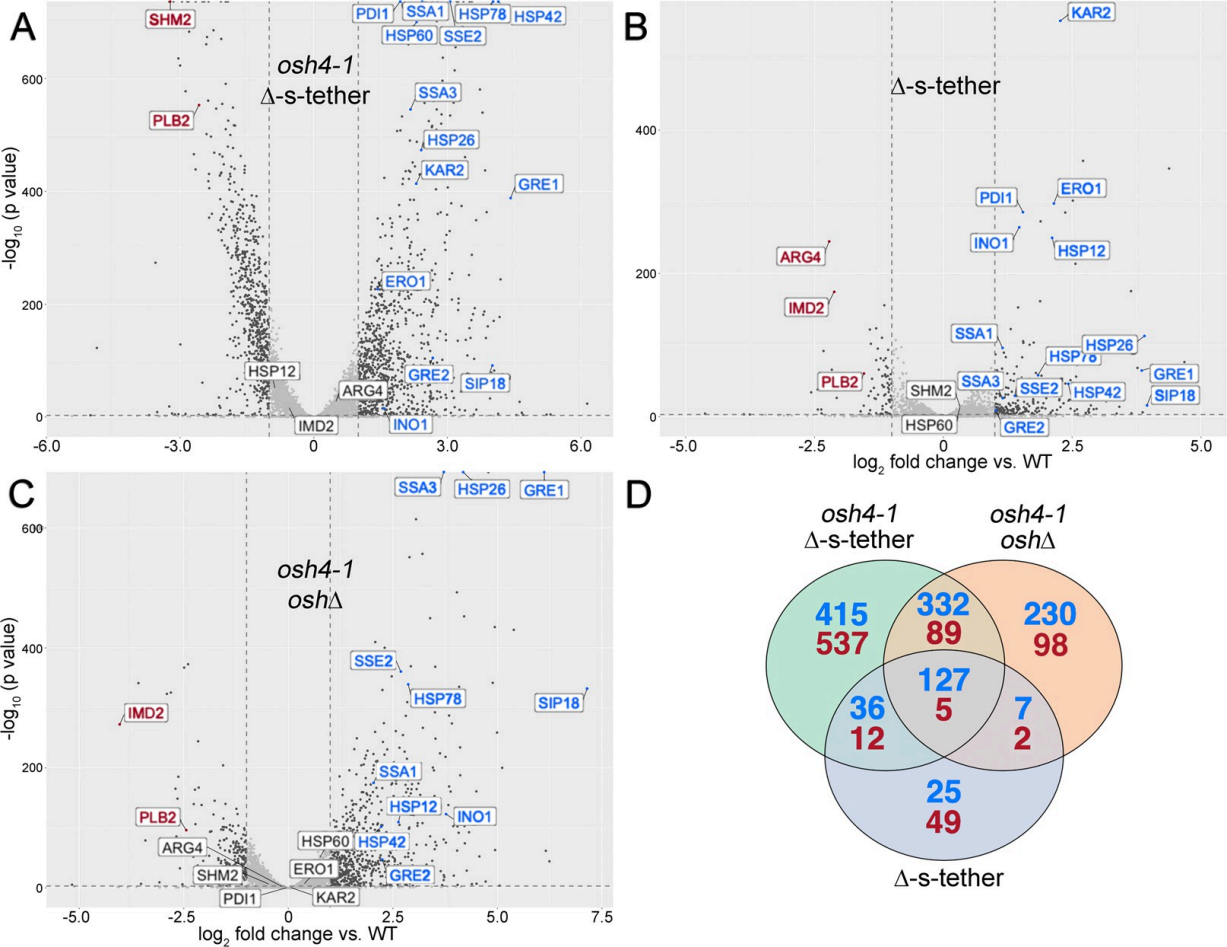
Transcriptomic profiles of *osh4-1*^*ts*^ Δ-s-tether, Δ-s-tether and *osh4-1*^*ts*^ *osh*Δ cells. Volcano plots showing relative transcript abundance in (**A)** *osh4-1*^*ts*^ Δ-s-tether (CBY6031), (**B)** Δ-s-tether (CBY5898) and (**C)** *osh4-1*^*ts*^ *oshΔ* (CBY926) cells grown in synthetic minimal media at 37°C. Plots show log_2_-fold expression change relative to WT (SEY6210) versus the negative log_10_-P value (y-axis). Transcript changes log_2_ ≥ or ≤ 1 are shown in black whereas representative stress pathway genes are blue and red, corresponding to induction or repression, respectively. (**D)** Venn Diagram showing overlapping subsets of upregulated (blue) or downregulated genes (red) in *osh4-1*^*ts*^ Δ-s-tether, Δ-s-tether and *osh4-1*^*ts*^ *oshΔ* cells.

We hypothesized that defects in specific regulatory pathways might underlie the lethality of *osh4-1*^ts^ Δ-s-tether cells, and those pathways might be identified by analyzing the genomic expression profile of *osh4-1*^ts^ Δ-s-tether cells. After incubation at 37°C for 1 h, isolated transcripts from WT and *osh4-1*^ts^ Δ-s-tether were compared by RNA deep sequencing analysis (RNA-seq) to determine genome-wide differences in transcript levels (Fig 4). To summarize, distinct transcriptome changes included genes implicated in ER and PM membrane stress (i.e. UPR, ER-associated protein degradation [ERAD], and HOG pathways), and changes in inositol regulation and lipid biosynthetic genes were also evident, as well as a general inhibition of metabolic, transcriptional, ribosomal/translational genes (Figs S2 and 4–7). Together these responses represent signature changes in the integrated regulatory pathway called the Environmental Stress Response (ESR) (Fig 6) [52,53].

**Fig 5:**
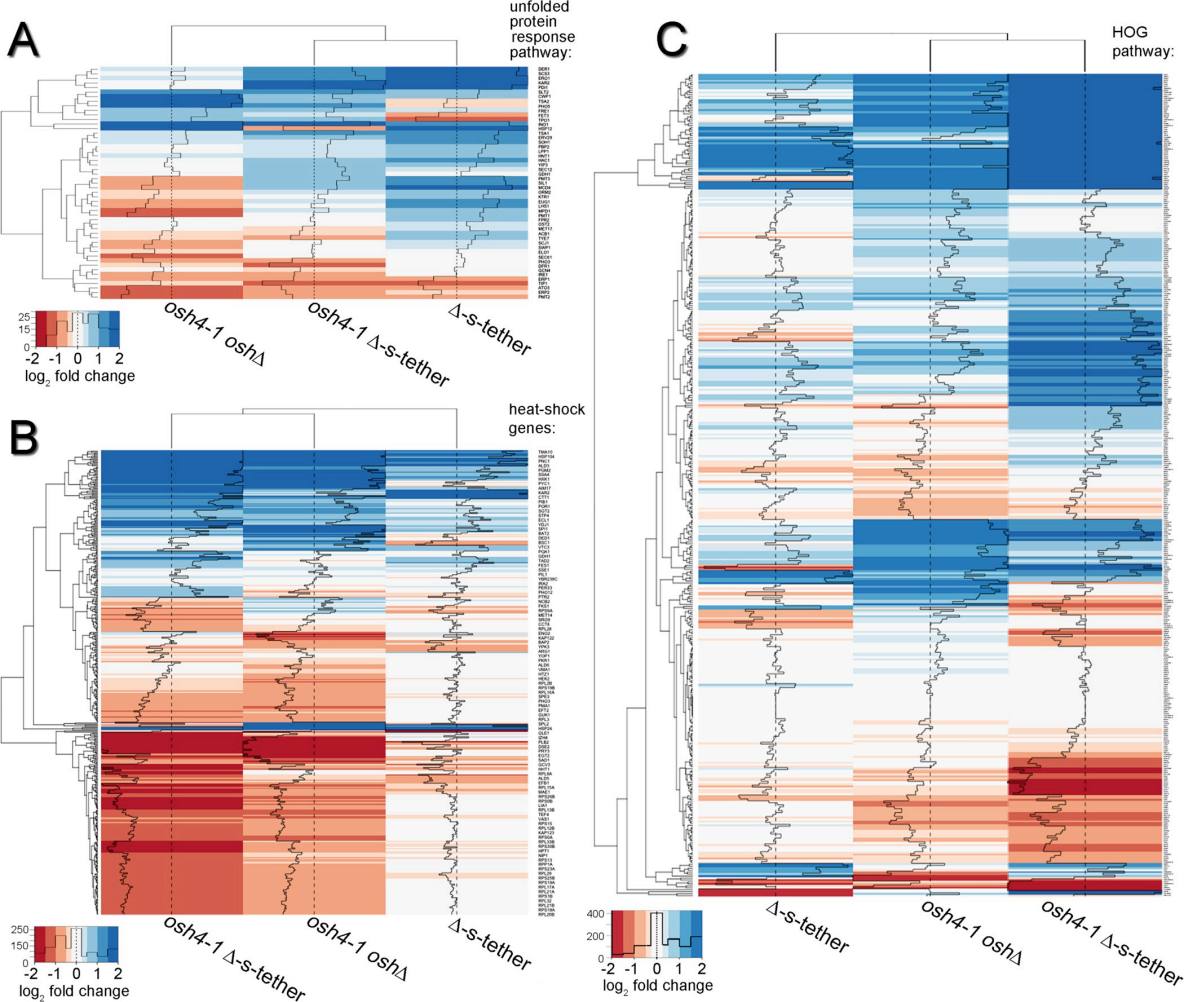
Effects of *osh4-1*^*ts*^ *osh*Δ, Δ-s-tether, and *osh4-1*^*ts*^ Δ-s-tether mutations on membrane stress pathways. Heatmap analyses of transcriptional responses relative to WT (SEY6210) at 37°C for 1 h in *osh4-1*^*ts*^ Δ-s-tether (CBY6031), Δ-s-tether (CBY5898), and *osh4-1*^*ts*^ *oshΔ* (CBY926) cells affecting **(A)** UPR, (**B)** heat shock and (**C)** high-osmolarity glycerol (HOG) pathway genes. Downregulated genes are shown in red, upregulated genes are shown in blue. UPR-responsive genes were curated from Kimata et al. [86] and using the Saccharomyces Genome Database (SGD), heat shock genes were curated from Hsf1p target genes and HOG pathway genes compiled from listed target genes of Msn2p/4p, Smp2p, Hot1p, and Sko1p. Histograms within each column represent the number of transcripts for each color in the heatmap.

**Fig 6:**
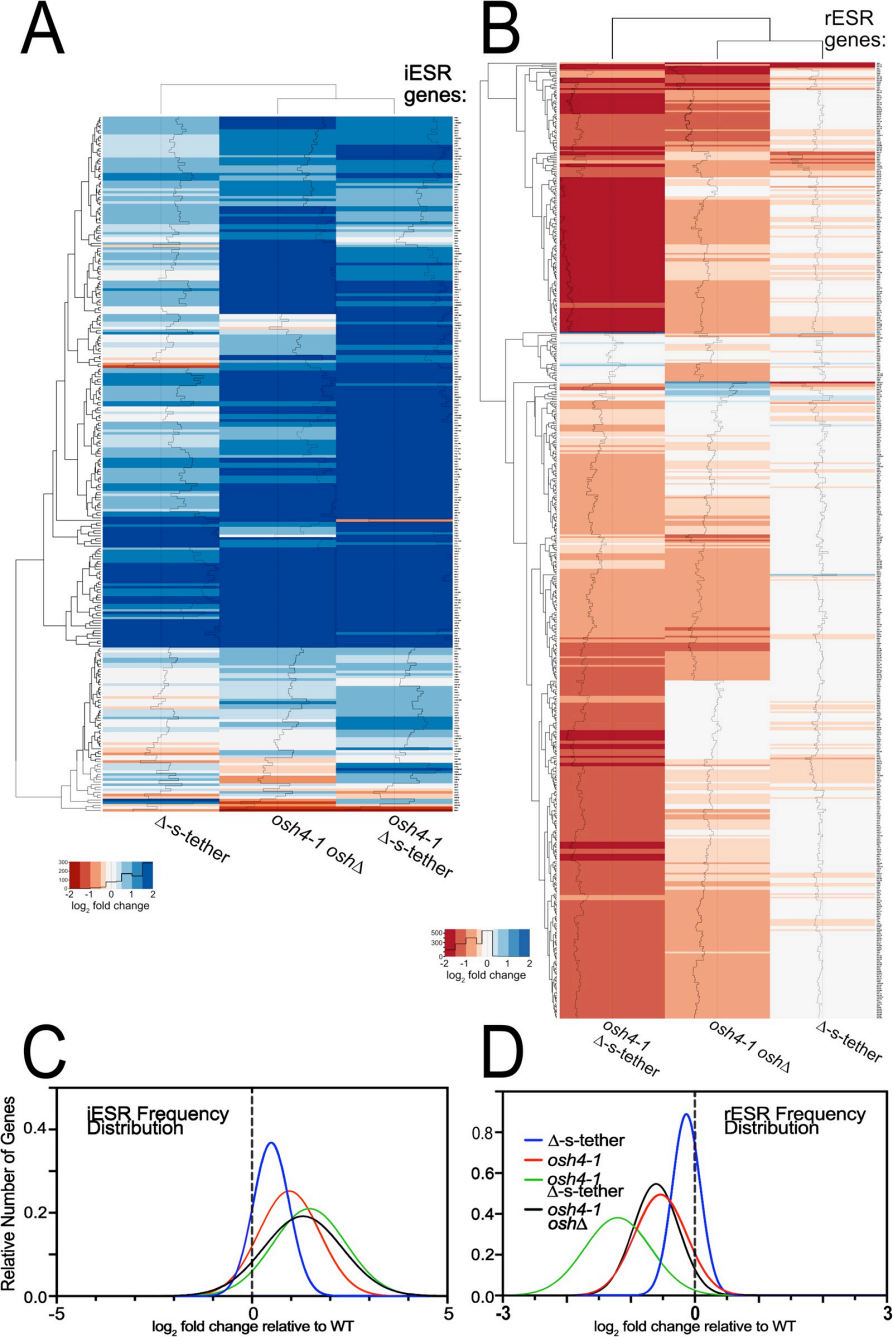
ESR responses to *OSH* and ER-PM tether mutations. Heatmap analyses of transcriptional responses relative to WT (SEY6210) at 37°C for 1 h in *osh4-1*^*ts*^ Δ-s-tether (CBY6031), Δ-s-tether (CBY5898), and *osh4-1*^*ts*^ *oshΔ* (CBY926) cells affecting **(A)** iESR and (**B)** rESR genes. Downregulated genes are shown in red, upregulated genes are shown in blue. iESR and rESR regulated genes were curated from previous reports [51,87]. Histograms within each column represents the number of transcripts for each color in the heatmap. **(C)** Graphical representations of the distribution of iESR and **(D)** rESR gene responses in *osh4-1*^*ts*^ Δ-s-tether, Δ-s-tether, and *osh4-1*^*ts*^ *oshΔ* cells.

**Fig 7:**
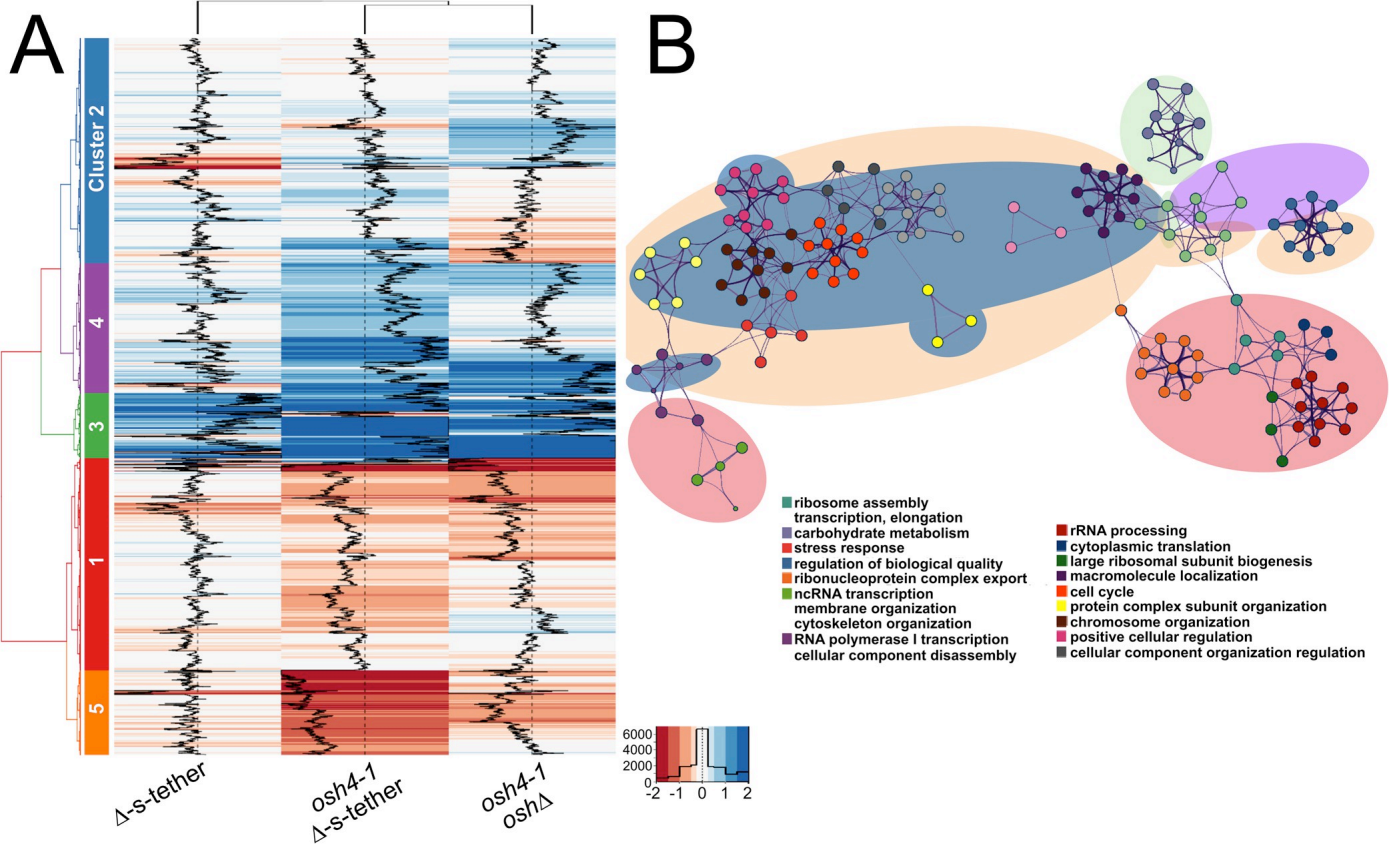
Hierarchical clustering of transcriptomic responses in *osh4-1*^ts^ *osh*Δ, Δ-s-tether, and *osh4-1*^ts^ Δ-s-tether cells. **(A)** Hierarchical clustering analysis of genomic transcriptional changes corresponding to *osh4-1*^*ts*^ Δ-s-tether (CBY6031) and *osh4-1*^*ts*^ *oshΔ* (CBY926) cells relative to WT (SEY6210) at 37°C for 1 h, and Δ-s-tether (CBY5898) relative to WT cultured at 30°C. Based on differential gene expression, genomic responses were separated into five distinct clusters, as denoted by the colours and numbers adjacent to the heatmaps and analyzed using Metaspace. **(B)** Clustered Gene Ontology (GO) analysis of gene functions corresponding to each of the five gene clusters. As specified by colour and described in the figure key, each node represents a distinct enrichment of gene sets that reflect a separate GO term. Clusters of nodes are grouped as corresponds to the clusters as shown in (A).

Specifically, in *osh4-1*^ts^ Δ-s-tether cells after 1 h at 37°C, transcript levels of 910 genes increased more than twofold (log_2_ ≥ 1) whereas 643 genes were downregulated twofold or less (log_2_ ≤ 1) as compared to the WT control (Fig 4A). Induced transcripts included regulatory categories enriched in heat-shock (e.g. *SSA1*, *SSA3*, *HSP60*, *SSE1*), UPR (e.g. *KAR2*, *PDI1*), and osmotic stress responses (e.g. *GRE1*, *GRE2*, *SIP18*) as well as key phospholipid synthetic genes (e.g. *INO1*); repressed transcripts represented specific lipid and general metabolism genes (e.g. *PLB1*, *SHM2*, *CDC60*), as well as transcriptional and translational genes (e.g. *RPA34*, *RPA135*, *RLP24*, *NOP4*) (Figs S2, 4A, 5, and 7). RNA-seq assay results were confirmed based on qPCR analysis of 3 upregulated genes (*SIP18*, *GRE2*, and *KAR2*) (S3 Fig).

Relative to WT gene expression, all genomic changes in *osh4-1*^ts^ Δ-s-tether cells after a 1 h incubation at 37°C, can be grouped into five distinct gene clusters (Fig 7). Two of these clusters contain induced ESR (iESR) genes, whereas most repressed ESR (rESR) genes are consolidated within two other clusters. iESR activation provides stress defense against multiple environmental stresses, including those that induce UPR and HOG membrane stress pathways [52,53]. rESR genes are involved in translation and ribosomal biosynthesis. rESR gene repression removes these highly expressed mRNAs from the translation pool to redirect translation toward iESR transcripts to hasten their expression and remedial responses [53]. Clustering analysis shows that 24.8% of cluster 3 (131 of 529 genes) and 9.9% of cluster 4 (105 of 1062) genes are associated with iESR, whereas 53.2% of cluster 5 (368 of 692 genes) and 9.9% of cluster 1 (171 of 1727 genes) are associated with rESR (Fig 7). Changes in iESR and rESR gene expression in *osh4-1*^ts^ Δ-s-tether cells represents a systemic cellular stress that is a consequence of disrupting integrated functions of the ER and PM.

### Distinct membrane stress pathways trigger ESR genes in cells lacking *OSH* or ER-PM tether genes

To assess how *OSH* and ER-PM tether genes contribute to the *osh4-1*^ts^ Δ-s-tether transcriptome profile, the profiles of cells lacking *OSH* genes were compared to those without ER-PM tethers. Using RNA-seq to establish the individual expression profiles of *osh4-1*^ts^ *osh*Δ and Δ-s-tether cells (Fig 4B and 4C), we hypothesized that together they would reflect the composite profile observed in *osh4-1*^ts^ Δ-s-tether cells. Because of the functional overlap of the entire *OSH* gene family, the *osh4-1*^ts^ allele alone was predicted to have little impact on genomic expression as compared to WT [28]. Despite the *OSH* genetic redundancy, *osh4-1* cells incubated at 37°C for 1 h elicit appreciable ESR gene changes as shown by RNA-seq, as compared to WT. The ESR gene changes mirror the transcriptomic profile of cells lacking all functional *OSH* genes, though by itself the *osh4-1* allele has more pronounced effects on rESR expression than iESR (S4 Fig). As such, we used the more robust *osh4-1*^ts^ *osh*Δ RNA-seq profile in most genomic comparisons. In fact, the prediction that the *osh4-1*^ts^ Δ-s-tether expression profile contains elements of both *osh4-1*^ts^ *osh*Δ and Δ-s-tether profiles was partially affirmed; over half of the induced genes, and about a fifth of repressed genes, corresponded to overlapping transcriptome changes detected in either *osh4-1*^ts^ *osh*Δ or Δ-s-tether cells, or both (Fig 4D).

When *OSH* genes are inactivated in *osh4-1*^ts^ *osh*Δ cells after 1 h at 37°C, major changes in iESR and rESR gene expression include gene categories affecting heat-shock (e.g. *SSA1*, *SSA3*, *SSE2*, *HSP12*, *HSP26*, *HSP78*) and osmotic stress MAP kinase (e.g. *GRE1*, *SIP18*) pathways, as well as global metabolism and ribosome biogenesis (Figs S2 and 4C). As indicated by the lack of UPR or ERAD gene changes, ER stress pathways are unaffected in *osh4-1*^ts^ *osh*Δ cells (Figs 4C and 5A). After 1 h at 37°C, 696 different gene transcripts increased more than twofold in *osh4-1*^ts^ *osh*Δ cells whereas 194 genes were downregulated twofold or less, relative to WT (Fig 4D). All told, the transcriptomic profile indicates that the ESR response to *OSH* gene inactivation primarily involves PM and cytoplasmic stress responses, as opposed to those affecting ER-related stress.

In Δ-s-tether cells, the transcriptomic profile also includes ESR genes with notable changes in UPR-induced genes (e.g. *KAR2*, *PDI1*, *ERO1*), heat-shock (e.g. *HSP12*) as well as phospholipid (e.g. *INO1*, *PLB2*), secondary metabolite and amino acid biosynthesis genes (Figs S2 and 4B). The constitutive activation of UPR genes is consistent with a previous report that analyzed cells bearing most of the same ER-PM tether gene deletions that are disrupted in the Δ-s-tether strain [2]. In Δ-s-tether cells, UPR induction specifically contributed to the broader iESR gene response and many repressed genes corresponded to rESR genes (Fig 6). These findings suggest that the elimination of ER-PM tethers elicits the ESR pathway, which is largely driven by constitutive UPR activation due to ER stress.

The expression profiles of *osh4-1*^ts^ *osh*Δ and Δ-s-tether cells share many ESR changes in gene expression, despite that fact the two strains have completely different effects on ER-PM MCS. We hypothesized that the contrasting differences on the amount of ER-PM MCSs in these strains might be reflected in their genomic profiles when analyzed for anticorrelation among gene expression clusters. Indeed, gene expression changes in genes clusters representing ER or PM functions were generally opposite for *osh4-1*^ts^ *osh*Δ versus Δ-s-tether cells (S5 Fig). In *osh4-1*^ts^ *osh*Δ cells, genes associated with PM function are generally upregulated whereas these genes are downregulated in Δ-s-tether cells. In Δ-s-tether cells, expression of ER-associated genes is upregulated but in *osh4-1*^ts^ *osh*Δ cells these genes are downregulated (S5 Fig). Transcriptomic profiling analysis suggests that the loss of ER-PM MCSs in Δ-s-tether cells activates ER-related gene expression while the gain of ER-PM MCSs resulting from *OSH* elimination results in transcriptional induction of PM-related responses. We propose that either drastic loss or gain of ER-PM MCSs triggers the integrated stress responses embodied by the ESR pathway. Of course, *OSH* gene deletion might have an impact on the ESR pathways due to other defects independent from ER-PM MCS increases.

### 5. Inositol attenuates UPR induction in Δ-s-tether cells indicative of Opi1p-dependent phospholipid gene transcriptional defects

Given that changes in lipid metabolism can induce UPR and ERAD ER stress response pathways [54], we hypothesized that UPR induction in Δ-s-tether cells might be due to their defects in phospholipid regulation [3]. As an important phosphoinositide precursor and regulator of phospholipid biosynthesis genes, inositol also affects Ire1p-dependent UPR induction [38,41,55–57]. To test if UPR activation in Δ-s-tether cells is contingent on inositol, we compared the transcriptomic profile of Δ-s-tether cells in the presence and absence of inositol. When inositol is removed from the culture medium, 195 gene transcripts increase twofold or more in Δ-s-tether cells and 68 genes are downregulated twofold or less (S6A Fig). Gene expression comparisons show that when cultured with inositol, constitutive UPR induction in Δ-s-tether cells is attenuated (S6A Fig). To determine if these inositol-dependent transcriptional changes affect cell growth, Δ-s-tether cells were cultured on solid growth media supplemented with and without inositol. However, exogenous inositol has a minimal effect on Δ-s-tether cell growth, though at 37°C the combination of inositol and choline decreases Δ-s-tether cell growth (S6C Fig). Regardless, in cells lacking ER-PM MCSs the inositol-dependent inhibition of UPR genes, and concomitant changes in phospholipid gene expression, suggests a response to ER membrane stress.

**Fig 8:**
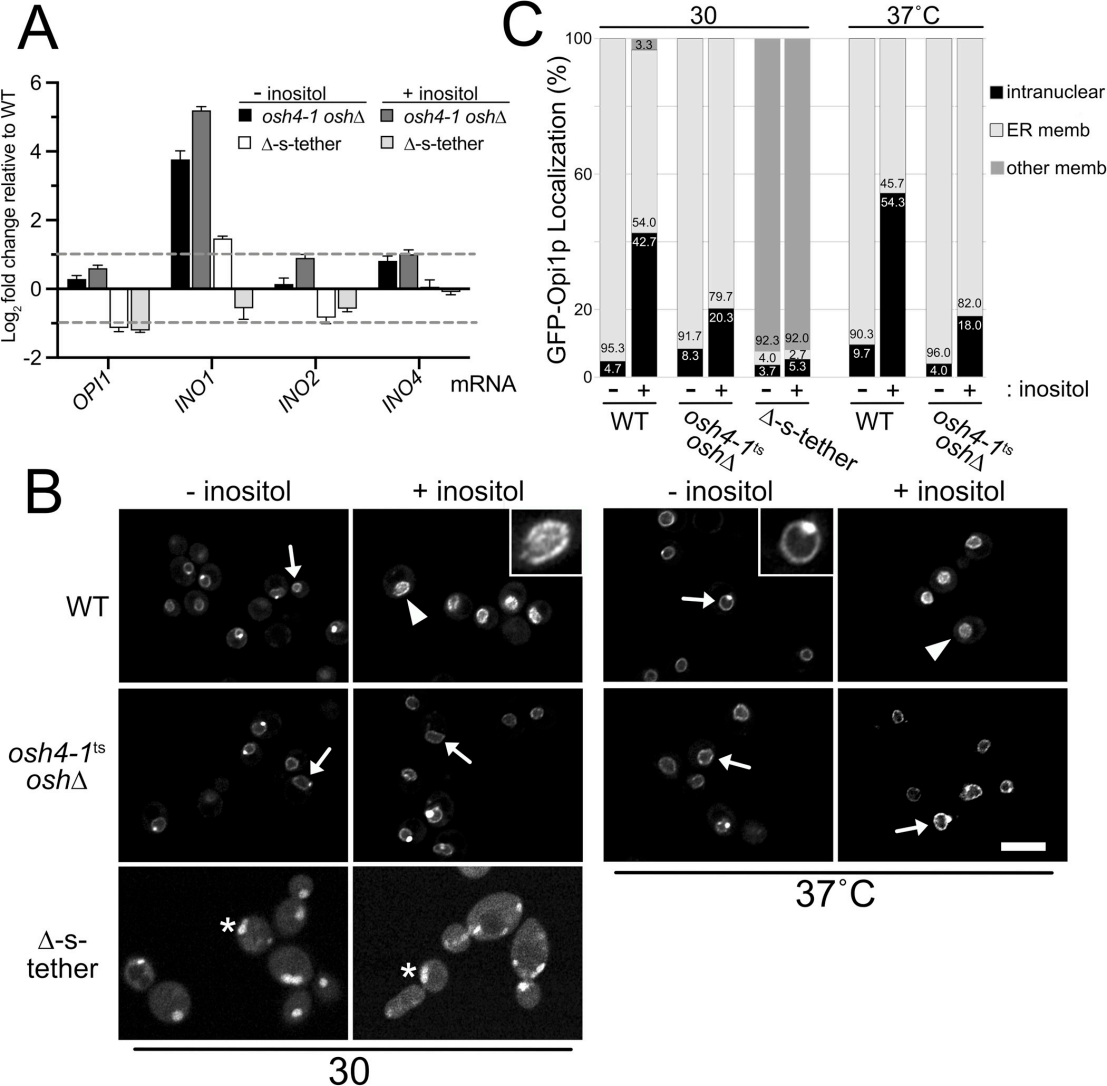
Phospholipid gene dysregulation due to Opi1p nuclear translocation defects. (A) Relative to WT (SEY6210), transcript abundance of *OPI1*, *INO1*, *INO2* and *INO4* in Δ-s-tether (CBY5838) cells cultured with or without 75 μM inositol at 30°C for 1 h. Relative mRNA levels of these phospholipid regulatory genes were also determined in *osh4-1*^*ts*^ *oshΔ* (CBY926) cells at 37°C for 1 h, with or without inositol. (B) Representative confocal fluorescent microscopy images of WT, *osh4-1*^*ts*^ *oshΔ*, and Δ-s-tether cells expressing GFP-Opi1p (pRS416-PHO5-GFP-OPI1) grown with or without 300 μM inositol at the growth temperatures indicated. Arrows indicate examples of GFP-Opi1p at the ER/nuclear membrane, arrowheads indicate intranuclear localization, and asterisks indicate examples of GFP-Opi1p localization distinct from either nuclear ER or intranuclear fluorescence. Inserts show examples of magnified images of nuclear membrane or intranuclear GFP-Opi1p. Scale bar = 5 μm. (C) Quantification of GFP-Opi1p localization in cells corresponding to panel (B). In response to inositol addition, the percentage of cells with GFP-Opi1p localization in the ER membrane, intranuclear, or other cellular membrane is indicated as shown (n = 300 cells per each strain).

Inositol restricted UPR induction causes changes in the Δ-s-tether genomic transcriptional profile but the *osh4-1*^ts^ *osh*Δ profile shows other changes in response to inositol addition (S6A and S6B Fig). However, transcription of some key phospholipid biosynthetic genes under control of the inositol-sensitive upstream activating sequence (*UAS*_*INO*_) are affected in *osh4-1*^ts^ *osh*Δ cells, Δ-s-tether and *osh4-1*^ts^ Δ-s-tether cells (Figs S2B and 4A–4C). In the absence of inositol, *UAS*_*INO*_ genes are normally targeted and activated by the master lipid biosynthetic transcription factors Ino2p and Ino4p [58,59]. In the presence of inositol, the Opi1p repressor is released from the ER membrane and translocates into the nucleus where it represses transcription of *UAS*_*INO*_ genes. Opi1p binding to the ER membrane is dependent on Scs2p and phosphatidic acid (PA) [59,60]. Within the nucleus, *INO1* (encoding inositol-3-phosphate synthase) represents the canonical *UAS*_*INO*_ regulated gene. In *osh4-1*^ts^ *osh*Δ, Δ-s-tether and *osh4-1*^ts^ Δ-s-tether cells, Opi1p-dependent *INO1* transcription is clearly dysregulated (Figs 4A–4C and 8A).

Because Δ-s-tether cells inherently lack Scs2p and have reduced levels of PA [3], the prediction is that Opi1p in these cells cannot be recruited to the ER/nuclear membrane and would only reside within the nucleus. In turn, this predicts the continuous intranuclear presence of Opi1p leading to *INO1* repression, with or without inositol. Relative to WT, however, *INO1* is highly expressed in the absence of inositol in Δ-s-tether cells, and *INO1* expression is near normal or slightly more repressed upon inositol treatment (Figs S5A, S5B, and 8A). To test if Opi1p mislocalization is the basis for this *INO1* dysregulation, Opi1p-GFP localization was visualized by fluorescence microscopy in response to inositol addition (Fig 8B). In WT cells cultured in the presence of 300 μM inositol for 1 h, most Opi1p-GFP is intranuclear whereas Opi1p-GFP is sequestered to the nuclear membrane/ER in the absence of inositol. In Δ-s-tether cells, however, Opi1p-GFP is neither intranuclear nor at the ER/nuclear membrane, with or without inositol (Fig 8B and 8C). Instead, Opi1p-GFP is observed on distinct cytoplasmic puncta consistent with lipid droplets and other non-nuclear compartments. While this finding explains even greater *INO1* expression relative to WT in the absence of inositol, *INO1* expression is near normal in Δ-s-tether cells in the presence of inositol (Fig 8A). Apart from Opi1p-GFP mislocalization, the transcription of both *OPI1* and the *INO2* activator is reduced, which might offset each other to provide the relatively normal *INO1* expression upon inositol addition (Fig 8A).

With or without inositol, in *osh4-1*^ts^ *osh*Δ cells *INO1* is constitutively expressed at high levels, suggesting that *INO1* regulation is completely uncoupled from Opi1p control (Fig 8A). As analyzed by confocal fluorescence microscopy, Opi1p-GFP is always localized to the nuclear membrane in *osh4-1*^ts^ *osh*Δ cells after incubation at 37°C for 1 h, regardless of inositol addition (Fig 8B and 8C). In cells lacking Osh proteins, inositol dependent Opi1p translocation from the ER membrane into the nucleus is disrupted. Although details in the mechanism may differ, phospholipid gene dysregulation in Δ-s-tether and *osh4-1*^ts^ *osh*Δ cells both involve defective Opi1p translocation into the nucleus.

### *OSH* and ER-PM tether gene expression is interdependent

Given the post-transcriptional induction of Tcb3p in *osh4-1*^ts^ *osh*Δ cells, we determined if *OSH* and ER-PM tether genes also impact one another on a transcriptional level. From the RNA-seq profile of Δ-s-tether cells, most *OSH* transcripts are unaffected relative to WT though *OSH1* transcription is significantly reduced (with or without inositol), *OSH5* transcription is decreased in the presence of inositol and increased in its absence, and *OSH6* transcription is activated in the absence of inositol (S7A Fig). In *osh4-1*^ts^ *osh*Δ cells incubated at 37°C for 1 h, the transcription of almost all tether genes (except *SCS22*) decreased (Figs S7B and 2E). Given that ESR affects transcription, and *osh4-1*^ts^ *osh*Δ cells have the greatest impact on ESR, decreases in ER-PM tether transcripts are consistent with the global reduction in transcription during ESR.

We hypothesized that the lethality of combining *osh4-1*^ts^ with Δ-s-tether mutations might stem from an exacerbation of shared defects, but this specific combination of mutations might result in new defects that disrupts cell growth. Of the induced transcripts in *osh4-1*^ts^ Δ-s-tether cells after 1 h at 37°C, 415 represent genes that are unaffected in *osh4-1*^ts^ *osh*Δ or Δ-s-tether cells (Fig 4D). Of the repressed transcripts, 537 genes were uniquely affected in the *osh4-1*^ts^ Δ-s-tether expression profile. Uniquely affected genes were enriched in KEGG categories involving ribosome biogenesis and general metabolism (S2 Fig). Because iESR and rESR includes these gene categories, the *osh4-1*^ts^ Δ-s-tether expression profile appears to represent a synergistic exacerbation of shared ESR defects, as opposed to causing new defects involving other pathways. Indeed, *osh4-1*^ts^ *osh*Δ, Δ-s-tether and *osh4-1*^ts^ Δ-s-tether expression profiles exhibit a moderate correlation, though the *osh4-1*^ts^ Δ-s-tether profile correlates most closely with that of *osh4-1*^ts^ *osh*Δ (S8 Fig). Based on the transcriptomic analysis, *OSH* gene inactivation appears to affect iESR by eliciting PM and cytoplasmic stress, whereas the elimination of tethers impacts iESR by inducing ER stress pathways. These responses suggest that the combination of *osh4-1*^ts^ and Δ-s-tether mutations elicits both PM and ER stresses, which together lead to a lethal synergistic exacerbation of ESR defects.

### HOG and UPR stress pathways induce Tcb3p-dependent ER-PM MCSs

**Fig 9:**
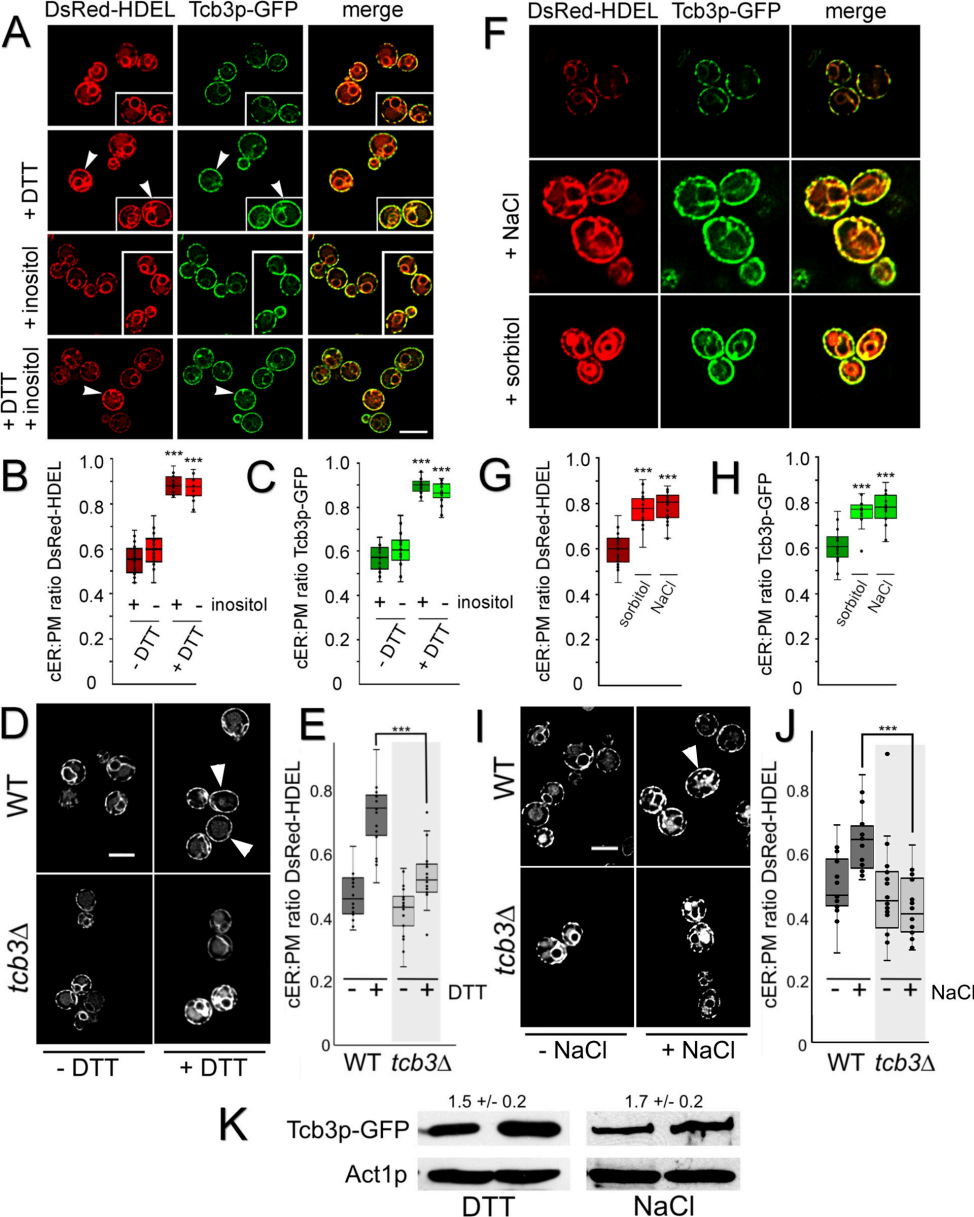
Both ER UPR and PM osmotic stresses increase Tcb3p-dependent ER-PM MCSs. **(A)** Representative fluorescent microscopy images of WT cells (CBY6087) expressing DsRed-HDEL (pRS416-DsRed-HDEL) and *TCB3*-GFP. Cells grown in synthetic minimal media with or without 75 μM inositol were treated with 2 mM DTT at 30°C for 2 h. (**B)** Ratios of the total length of cortical DsRed-HDEL fluorescence (cER) per total PM in WT, and (**C)** Relative ratios of cortical Tcb3p-GFP fluorescence per PM in WT cells, corresponding to A (n = 20 cells per condition). (**D**) Representative images of WT (BY4742) and *tcb3*Δ (CBY7354) cells expressing DsRed-HDEL treated with 2 mM DTT at 30°C for 2 h. (**E**) Quantification of WT and *tcb3*Δ cER per total PM corresponding to D (n = 20 cells per strain). (**F)** Representative fluorescent microscopy images of WT (CBY6087) cells expressing DsRed-HDEL (pRS416-DsRed-HDEL) and *TCB3*-GFP. Cells were cultured in synthetic minimal media with or without 0.7 M NaCl or 1 M sorbitol at 30°C for 2 h. (**G)** Relative ratios of cortical DsRed-HDEL fluorescence (cER) per PM in WT, and (**H)** Ratios of cortical Tcb3p-GFP fluorescence per PM in WT, corresponding to F (n = 20 cells per condition). (**I**) Representative images of WT (BY4742) and *tcb3*Δ (CBY7354) cells expressing DsRed-HDEL treated with 0.7 M NaCl at 30°C for 2 h. (**J**) Quantification of WT and *tcb3*Δ cER per total PM corresponding to I (n = 20 cells per strain). (**K**) Representative immunoblot probed with anti-GFP and anti-actin antibodies showing relative GFP-Tcb3p levels in WT cells cultured with 2 mM DTT or 0.7 M NaCl for 2 h at 30°C, relative to the actin (Act1p) control (mean ± SD; n = 3). Arrowheads indicate cells with nearly absolute coverage of the cortex with ER or Tcb3p. Box and whisker plots and statistics as described in Fig 1. Statistical significances compare treated to untreated cells for panels B, C, G, and H, and treated WT versus treated *tcb3*Δ cells for E and J. ***p ≤ 2 x 10^−7^. Scale bars = 5 μm.

As shown in *OSH* and Δ-s-tether mutants, changes in ER-PM MCS correlate with HOG and UPR gene activation. We also tested the converse, namely, whether WT cells subjected to specific ER or PM membrane stresses induce changes in ER-PM MCSs. Low-dose treatments of yeast with dithiothreitol (DTT) induces the UPR, and mutants involved in the UPR pathway fail to grow when challenged with 2 mM DTT [36,38,55]. To test the effect of UPR induction on ER-PM association, the cortical localizations of both DsRed-HDEL and Tcb3p-GFP were analyzed in DTT-treated and inositol-starved WT cells (Fig 9A–9C). In WT cells, cortical DsRed-HDEL fluorescence increases from 56% to ~90% after a 2 h treatment with 2 mM DTT, and a 2 h treatment with 0.7 M NaCl results in a comparable increase. Inositol depletion caused statistically insignificant increases in either DsRed-HDEL or Tcb3p-GFP at the cortex (Fig 9A–9C). However, UPR activation by 2 mM DTT significantly increased the cortical colocalization of DsRed-HDEL and Tcb3p-GFP to where the PM was nearly completely covered with both (~90%; Fig 9A–9C). DTT induction of cortical ER-PM association was also inositol independent (Fig 9A–9C). Consistent with previous reports, *TCB3* deletion by itself has minimal effects on ER-PM association (Fig 9D and 9E) [2,5]. In response to DTT treatment, however, the extensive spread of ER along the cortex as observed in WT cells does not occur in *tcb3*Δ cells. DsRed-HDEL localization in DTT-treated *tcb3*Δ cells is comparable to untreated WT cells and significantly reduced compared to DTT-treated WT cells (Fig 9D and 9E). With or without supplemented inositol, UPR activation induces Tcb3p-dependent ER-PM MCS formation in WT cells.

We predicted that ER-PM MCSs would affect PM integrity as well as cortical ER structure, so we tested if stress conditions that perturb PM integrity also impact ER-PM MCSs. Osmolarity stresses on the PM can be induced by high salt concentrations or 1 M sorbitol in the growth medium [61]. WT cells expressing DsRed-HDEL and Tcb3-GFP were cultured for 2 h in hyperosmotic media containing either 0.7 M NaCl or 1 M sorbitol. Both these PM stresses increased DsRed-HDEL and Tcb3-GFP fluorescence and expanded cortical coverage to >75% of the PM (Fig 9F and 9H). These results confirmed the functional relevance of HOG pathway gene induction as revealed in *osh4-1*^ts^ *osh*Δ and *osh4-1*^ts^ Δ-s-tether transcriptomic profiles.

As with DTT induction of ER stress, PM stress also increases Tcb3p-dependent ER-PM association. In *tcb3*Δ cells treated with 0.7 M NaCl for 2 h, DsRed-HDEL localization is comparable to untreated WT cells but significantly reduced compared to NaCl treated WT cells (Fig 9I and 9J). Thus, *TCB3* is necessary for the induction of ER-PM association in response to NaCl and HOG pathway activation. In fact, both DTT and NaCl treatments increase Tcb3p expression as determined by immunoblot analysis. Tcb3p levels are 1.5–1.7 fold higher in WT cells treated with 4 mM DTT or 0.7 M NaCl for 2 h (Fig 9K). Both ER and PM stresses induce ER-PM MCSs by increasing Tcb3p levels.

### Osh proteins and ER-PM MCSs bolster UPR and HOG pathways

**Fig 10:**
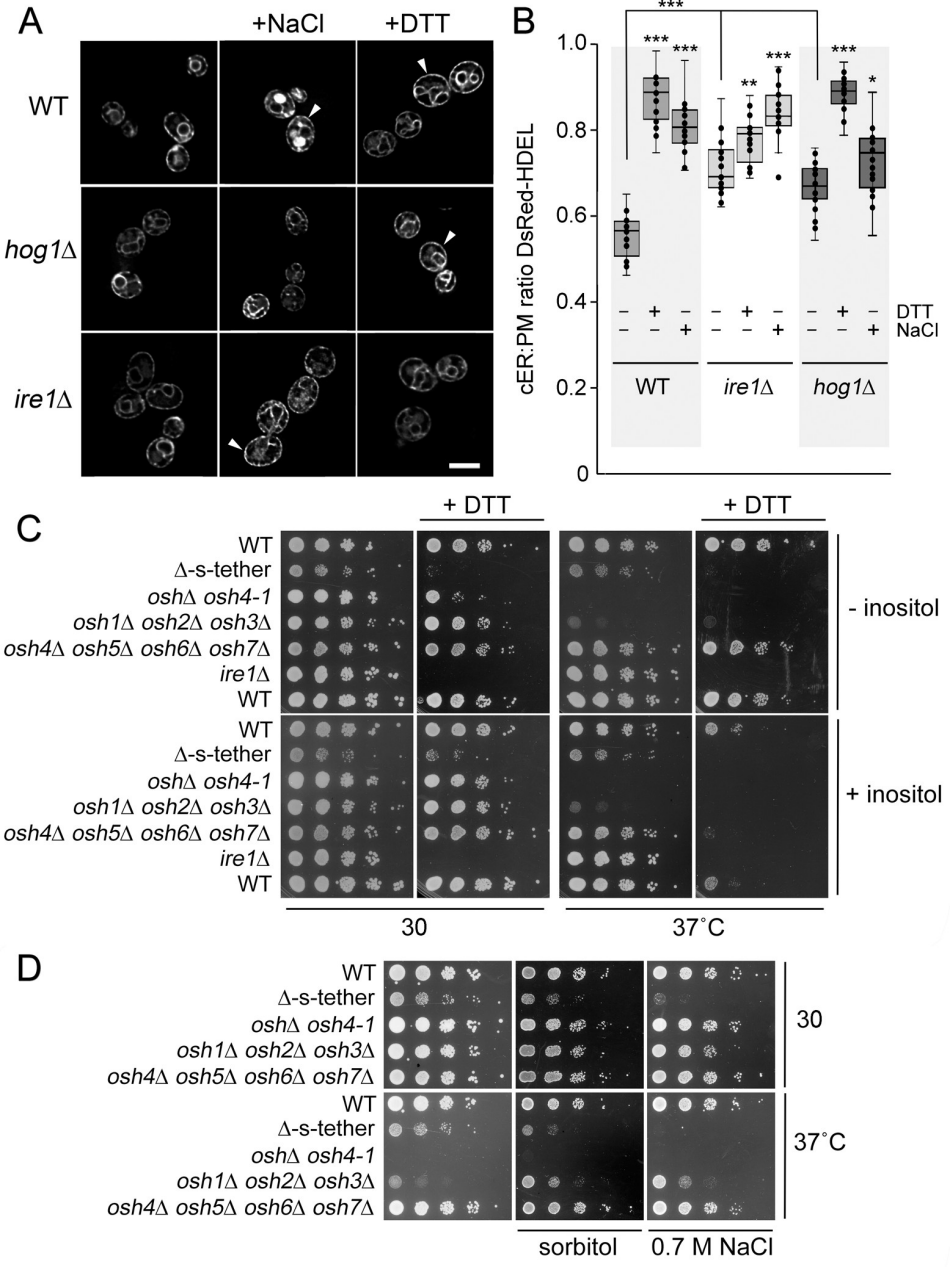
ER-PM MCS dependence on *IRE*1, *HOG1*, and *OSH*s. **(A)** Representative fluorescent microscopy images of WT (BY4741), *ire1Δ* (CBY1048), and *hog1Δ* (CBY6465) cells expressing DsRed-HDEL (pRS416-DsRed-HDEL). Cells cultured in synthetic complete media were treated with 2 mM DTT or 0.7 M NaCl at 30°C for 2 h. Arrowheads indicate examples of nearly complete association of ER with the cell cortex. (**B)** Ratios of the total length of cortical DsRed-HDEL fluorescence (cER) per total PM length in WT, *ire1Δ*, and *hog1Δ* cells (n = 20 cells for strain and each condition; statistical significance shown for treated versus untreated cells of the same genotype). (**C)** Tenfold serial dilutions of WT (SEY6210), Δ-s-tether (CBY5838), *osh4-1*^*ts*^ *oshΔ* (CBY926), *osh1Δ osh2Δ osh3Δ* (JRY6253), *osh4Δ osh5Δ osh6Δ osh7Δ* (JRY6272), *ire1Δ* (CBY1048) and WT (BY4741) cells spotted onto solid synthetic minimal media with or without 4 mM DTT or 75 μM inositol and cultured at 30°C or 37°C for 2–4 days. (**D)** Tenfold serial dilutions of WT, Δ-s-tether, *osh4-1*^*ts*^ *oshΔ*, *osh1Δ osh2Δ osh3Δ*, *osh4Δ osh5Δ osh6Δ osh7Δ* cells grown on solid synthetic minimal media with or without 0.7 M NaCl or 1M sorbitol at 30°C or 37°C for 3–5 days. Box and whisker plots and statistics as described in Fig 1. ***p ≤ 1.2 x 10^−7^, **p = 0.001, *p = 0.01. Scale bars = 5 μm.

The Hog1p and Ire1p kinases are key regulators of the HOG and the UPR pathways, respectively. Although both pathways operate independently, they are functionally linked [62]. To test if either pathway affects cortical ER-PM association, we eliminated *HOG1* and *IRE1*, the key regulatory kinases in PM and ER stress response pathways respectively. Relative to WT, cortical DsRed-HDEL somewhat increased in untreated *ire1*Δ and *hog1*Δ cells (Fig 10B and 10C). Unlike WT cells treated with DTT, however, *ire1*Δ cells exhibit only a minor additional increase in cortical DsRed-HDEL fluorescence after DTT treatment (Fig 10B and 10C). This result indicates that the *IRE1* deletion prevents increased cortical ER-PM association in response to ER stress. With respect to PM stress, *hog1*Δ cells only exhibited a modest 5% increase in PM-associated DsRed-HDEL when treated with 0.7 M NaCl for 1 h, which was far less relative to the increase in salt-treated WT cells. Increased ER-PM association in response to membrane stress is dependent on both *HOG1* and *IRE1*.

The HOG pathway has been shown to affect UPR activity [62]. However, *hog1*Δ cells had no growth sensitivity to 4 mM DTT and *ire1*Δ cells exhibited only minor growth sensitivity to 0.7 M NaCl, as compared to WT (S9 Fig). Moreover, cortical DsRed-HDEL was equivalent to WT cells in NaCl-treated *ire1*Δ cells and DTT-treated *hog1*Δ cells. Under the membrane stress conditions we tested, changes in cortical ER-PM association seem to reflect independent responses by UPR and HOG pathways, even if those responses are integrated in the ESR pathway.

We next tested how ER stress affects Δ-s-tether and *OSH* mutant cell growth. In the absence of inositol, both Δ-s-tether and *ire1*Δ cells are hypersensitive to DTT-induced ER stress at 30°C (Fig 10C). When inositol is included in the growth medium, however, Δ-s-tether cells grow better. In contrast, *osh1*Δ *osh2*Δ *osh3*Δ cells are largely unaffected by DTT at 30°C, and these cells do not grow at 37°C. Cells lacking the subset of small *OSH* genes (*osh4*Δ *osh5*Δ *osh6*Δ *osh7*Δ) do not exhibit DTT sensitivity in the absence of inositol (Fig 10C). We note that at 37°C, DTT together with inositol perturbed growth of all strains analyzed (including WT). Altogether, cells lacking ER-PM tethers are sensitive to ER stress, but UPR induction has differential effects on the long versus short *OSH* mutants, depending on presence of inositol.

Likewise, *HOG* pathway genes are also induced in cells lacking tethers or *OSH* genes. When treated with the hyperosmotic agents (0.7 M NaCl and 1 M sorbitol), WT and *OSH* mutant cells are largely unaffected, but Δ-s-tether cell growth is moderately perturbed (Fig 10D). As such, cells without ER-PM MCSs appear to be sensitive to both ER and PM stress, whereas *OSH* mutants are more affected by ER stress.

**Fig 11:**
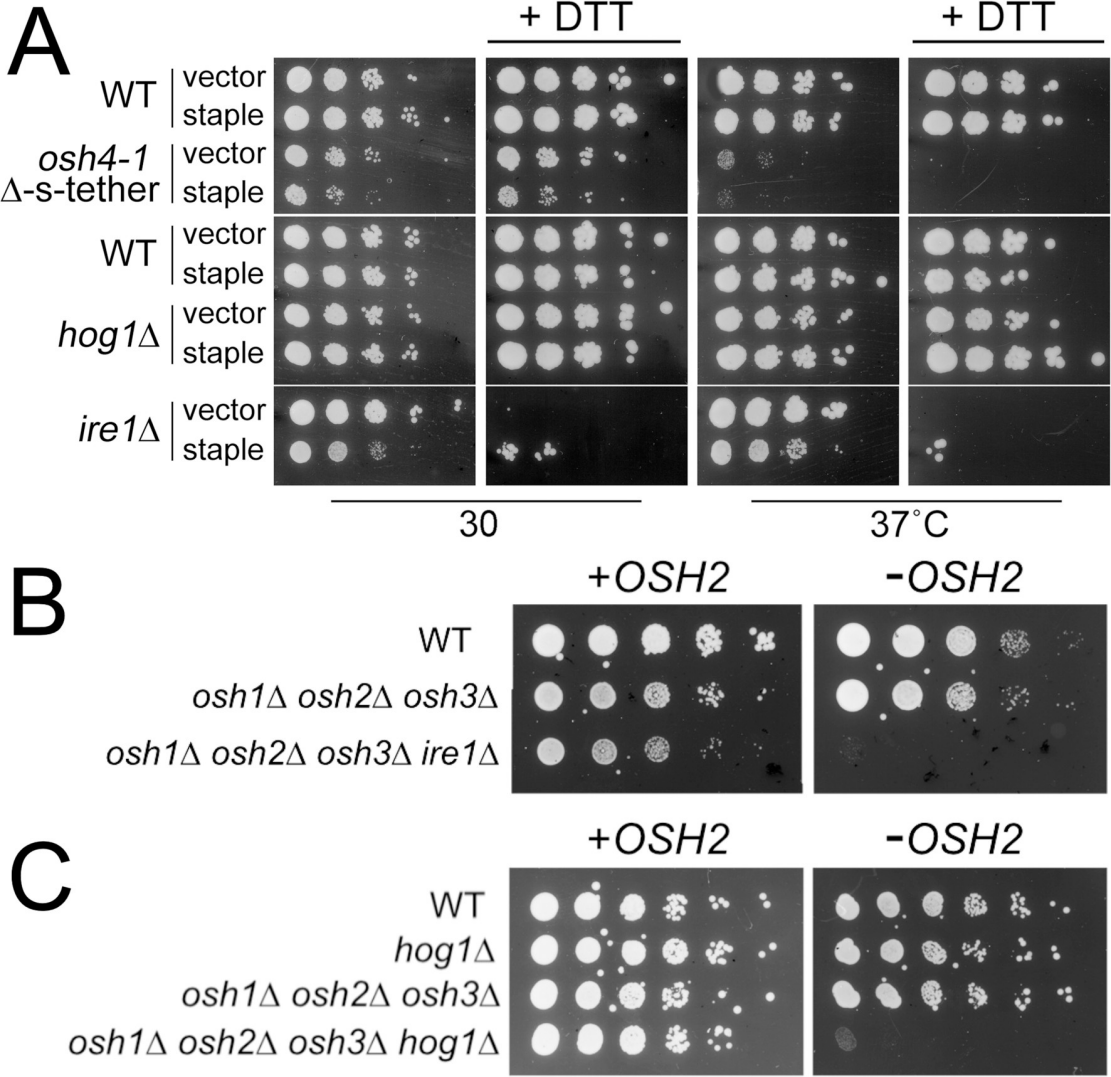
The “artificial ER-PM staple” rescues UPR inactivation, and deletion of *HOG1* or *IRE1* is synthetically lethal with *osh1Δ osh2Δ osh3Δ*. (**A)** Tenfold serial dilutions of *osh4-1*^*ts*^ Δ-s-tether cells with its congenic WT (SEY6210) control, and *hog1*Δ and *ire1*Δ with their congenic WT (BY4741) control, expressing either the “artificial membrane staple” (pCB1185) or the vector control (YCplac111). Cells were grown at 30°C or 37°C on synthetic medium with or without 4 mM DTT for 3 or 5 days, respectively. (**B)** Tenfold serial dilutions of WT (SEY6210), *osh1Δ osh2Δ osh3Δ* (JRY6263), and *osh1Δ osh2Δ osh3Δ ire1*Δ (CBY6916) cells containing an episomal copy of *OSH2* (+*OSH2*; pCB113). Cells were spotted onto selective solid medium, or on solid medium containing 5-FAA to select against the *OSH2* containing plasmid (-*OSH2*) and grown for 3 days at 30°C. Removal of the *OSH2* plasmid from *osh1Δ osh2Δ osh3Δ ire1*Δ cells is lethal. (**C)** Tenfold serial dilutions of WT (SEY6210), *hog1*Δ (CBY6912), *osh1Δ osh2Δ osh3Δ* (JRY62563), *osh1Δ osh2Δ osh3Δ hog1*Δ (CBY6914) cells that all contain an episomal copy of *OSH2* (+*OSH2*; pCB113). Cells were spotted onto selective solid media, with or without 5-FAA to select against the *OSH2* containing plasmid (-*OSH2*) and grown for 4 days at 30°C. The *OSH2* plasmid is necessary for *osh1Δ osh2Δ osh3Δ hog1*Δ cells growth indicating that *HOG1* deletion in *osh1Δ osh2Δ osh3Δ* cells is lethal.

To further test if MCSs help protect cells against PM or ER membrane stress, we generated cells with extra ER-PM MCSs as conferred by an artificial ER-PM staple [3]. Artificial ER-PM staples, which are hybrid tethers constructed from non-yeast protein domains, were expressed in WT cells to augment the normal complement of endogenous tethers without generally increasing ER membrane amounts. These cells were then challenged with hyperosmotic or ER stresses. In Fig 11A, the effect of additional ER-PM MCSs is shown on WT, *osh4-1*^ts^ *osh*Δ, *osh4-1*^ts^ Δ-s-tether, *ire1*Δ, and *hog1*Δ cells when challenged with DTT-induced ER stress. Under ER stress conditions that otherwise kill *ire1*Δ cells, the artificial staple restores limited growth (Fig 11A). Under hyperosmotic conditions (5 M NaCl), the artificial staple confers no significant growth advantage to *hog1*Δ cells or any other strains tested. Nonetheless, even nonspecific cortical ER-PM contact provides protection against ER stress.

These results suggested that ER-PM tethers and, specifically, the subset of long Osh proteins contribute to ER stress response. In support of these findings, the deletion of *IRE1* in *osh1Δ osh2Δ osh3Δ* cells results in lethality (Fig 11B). Although none of the *OSH* deletion mutants tested exhibited sensitivity to hyperosmotic agents, we also found that combining *hog1*Δ with *osh1Δ osh2Δ osh3Δ* mutations causes significant growth defects, even in the absence of any hyperosmotic condition (Fig 11C). The long *OSH* genes appear to connect the UPR regulation with the HOG pathway.

### *osh4-1*^*ts*^ Δ-s-tether lethality is suppressed by specific *OSH* genes and the lipid kinase *DGK1*

Although this study reports functional differences in *OSH* genes with respect to ER-PM MCSs, the entire *OSH* gene family shares an essential function in which any *OSH* gene can replace another [28]. As relating to ER-PM MCSs, increased gene dosage of *OSH6* rescues *osh4*Δ Δ-s-tether lethality [3]. To determine if this suppression is unique or shared by other *OSH* genes, we expressed each of the seven *OSH* genes on high-copy plasmids in *osh4-1*^ts^ Δ-s-tether cells (S10 Fig). As expected, high-copy *OSH6* rescues the lethality of *osh4-1*^ts^ Δ-s-tether cells at elevated temperatures. High-copy *OSH2* or *OSH4* poorly suppress *osh4-1*^ts^ Δ-s-tether temperature sensitivity. It should be noted that high-copy *OSH4* is not a robust suppressor likely because *OSH4* overexpression causes growth defects even in WT cells [22]. High-copy expression of most *OSH* genes, except for *OSH1*, *OSH6* and *OSH7*, have little impact on Δ-s-tether cells growth defects at 37°C (S10 Fig). Although the *OSH* gene family shares an essential function [25,30], only specific *OSH* genes suppress lethality in *osh4-1*^ts^ Δ-s-tether cells.

**Fig 12:**
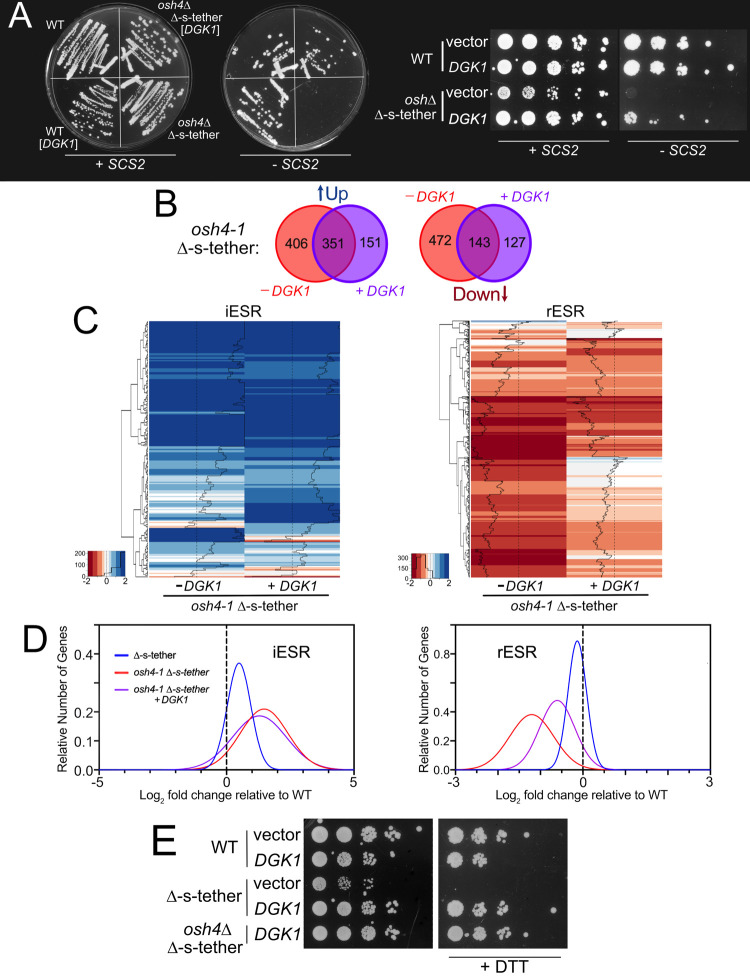
*DGK1* is a multicopy suppressor of *osh4* Δ-s-tether lethality that alleviates rESR gene repression. **(A)** Left: WT (SEY6210) and *osh4Δ* Δ-s-tether cells (CBY5988) that contain *SCS2* on a *URA3*-marked plasmid (pSCS2), transformed with a high-copy 2μ plasmid expressing *DGK1* (pCB1346) or a vector control (YEplac181), onto solid growth medium. Cells were cultured for 5 days at 30°C on growth medium (containing 5’-FOA) to select against the *SCS2*-containing plasmid (-*SCS2*). As compared to growth on standard synthetic medium (+*SCS2*), *osh4Δ* Δ-s-tether cells are only viable when *DGK1* was present without *SCS2*. Right: Similar to above, tenfold serial dilutions of WT and *osh4Δ* Δ-s-tether cells containing both the *SCS2* plasmid and high-copy *DGK1*, or the vector control, spotted on solid growth media with 5’-FOA (-*SCS2*) or without (+*SCS2*). High-copy *DGK1* rescues the lethality of *osh4Δ* Δ-s-tether mutations. (**B)** Venn Diagram indicating numbers of upregulated (↑UP) or downregulated genes (DOWN↓) in *osh4-1*^ts^ Δ-s-tether cells with (+ *DGK1;* purple) or without (- *DGK1;* light red) high-copy *DGK1* plasmids at 37°C for 1 h, relative to WT. **(C)** Heatmap analyses of iESR and rESR transcriptional responses in *osh4-1*^*ts*^ Δ-s-tether cells with or without high-copy *DGK1* expression relative to WT, at 37°C for 1 h. Downregulated genes shown in red; upregulated genes shown in blue. **(D)** Graphical representation of the distribution of iESR and rESR gene responses in *osh4-1*^*ts*^ Δ-s-tether, with and without high-copy *DGK1* expression. **(E)** Tenfold serial dilutions of WT and Δ-s-tether (CBY5838) cells transformed either with vector or high-copy *DGK1*, and *osh4*Δ Δ-s-tether cells suppressed with high copy *DGK1* (CBY6506). Cells were grown at 30°C on synthetic medium with or without 4 mM DTT for 4–5 days.

To identify essential functions disrupted in *osh4-1*^ts^ Δ-s-tether cells, extragenic suppressors were selected after *osh4-1*^ts^ Δ-s-tether cells were transformed with a high-copy (2μ) genomic library and cultured at 37°C. Of the extragenic suppressors obtained, we obtained 62 plasmids from a total of 5 genomic equivalents transformed. The most abundant extragenic suppressor genomic fragments (other than the expected *OSH* and tether genes) included the gene *DGK1* (isolated 12 times), which encodes a diacylglycerol kinase that synthesizes PA from diacylglycerol (DAG) [63]. When subcloned from a genomic library plasmid, the *DGK1* gene was confirmed as the suppressor of the original *osh4-1*^ts^ Δ-s-tether conditional mutant. High-copy *DGK1* is also a bypass suppressor given that it rescues *osh4*Δ Δ-s-tether lethality (Fig 12A). Other selected *osh4-1*^ts^ Δ-s-tether suppressors do not act as bypass suppressors and provide comparatively weak suppression.

To determine the genetic changes conferred by *DGK1* suppression, transcriptomic profiles of *osh4-1* Δ-s-tether cells with and without high-copy *DGK1* were compared. Of the 757 upregulated genes (log_2_ ≥ 1) in *osh4-1* Δ-s-tether cells, 53.6% are reduced by *DGK1* suppression while 76.7% of the 615 downregulated genes (log_2_ ≤ 1) increase towards WT levels (Fig 12B). Of course, *DGK1* overexpression itself elicits novel effects including the upregulation of 151 new genes (log_2_ ≥ 1) and 127 new downregulated genes (log_2_ ≤ 1) in *osh4-1* Δ-s-tether cells. Although *DGK1* overexpression in *osh4-1* Δ-s-tether cells has little impact on iESR gene activation, rESR gene expression trended closer to WT levels (Fig 12C and 12D). The functional consequence of *DGK1* suppression is also evident when challenging Δ-s-tether and *osh4*Δ Δ-s-tether cells with ER stress. As shown in Fig 12E, DTT sensitivity of Δ-s-tether strains is suppressed by *DGK1* overexpression. The mechanism of rescue of *osh4-1* Δ-s-tether lethality by *DGK1* appears to involve alleviating rESR inhibition of gene expression as well as assuaging sensitivity to ER stress.

**Fig 13:**
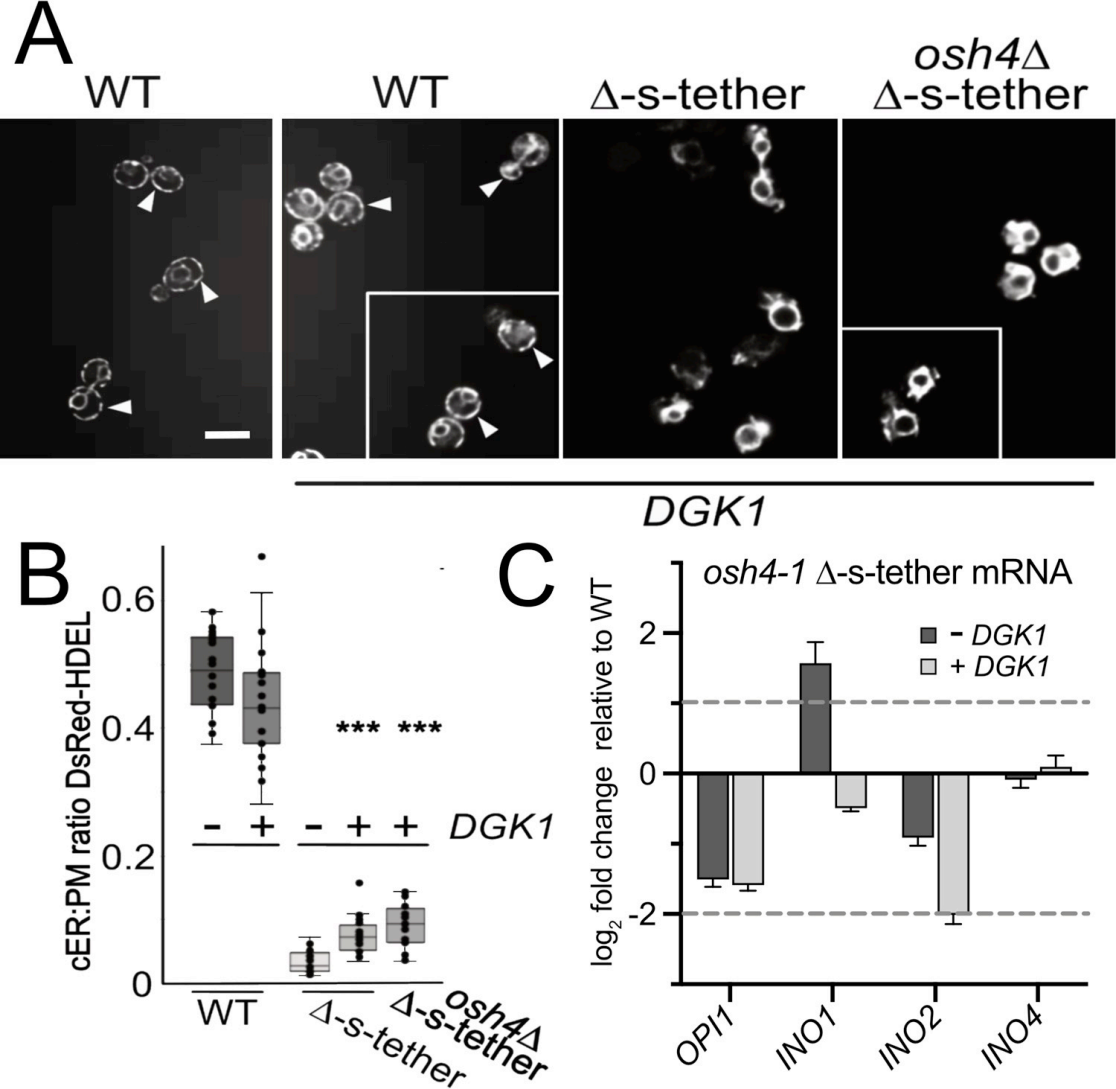
Representative fluorescent microscopy images of WT (SEY6210) and Δ-s-tether (CBY5838) cells both transformed with a control vector (YEplac181) or high-copy *DGK1* (pCB1346), *osh4Δ* Δ-s-tether cells suppressed with high-copy *DGK1* (CBY6506), all expressing DsRed-HDEL (pRS416-DsRed-HDEL). Cells were cultured in synthetic growth medium at 30°C. Arrowheads indicate examples of cortical ER association with the PM. (**B)** Ratios of the total length of cortical DsRed-HDEL fluorescence (cER) per PM in WT, Δ-s-tether, and *osh4Δ* Δ-s-tether cells with or without high-copy 2μ *DGK1* as indicated (n = 20 cells; statistical significance shown for Δ-s-tether versus Δ-s-tether and *osh4Δ* Δ-s-tether cells transformed with high-copy *DGK1*; ***p ≤ 3 x 10^−7^). Box and whisker plots and statistics as described in Fig 1. **(C)** Relative to WT, mRNA levels of *OPI1*, *INO1*, *INO2* and *INO4* in *osh4-1*^*ts*^ Δ-s-tether (CBY6031) cells transformed with the vector (- *DGK1*) or high-copy *DGK1* (+ *DGK1*) cultured at 37°C for 1 h.

With the artificial ER-PM staple, we showed increased attachment of cortical ER to the PM can occur without increasing cellular ER amounts [3]. In contrast, *DGK1* overexpression had been previously shown to generally expand ER throughout the cytoplasm [36,63]. As a formal possibility, we investigated if *DGK1*-induced proliferation of cytoplasmic ER might non-specifically drive extra ER toward the cell cortex, even without active ER tethering to the PM. In this scenario, enlarged cytoplasmic ER would stochastically push ER into close proximity with the PM perhaps fulfilling the role of ER-PM MCSs. Despite increases in cytoplasmic ER, *DGK1* overexpression did not lead to increased cortical ER in WT cells as previously reported (Fig 13A and 13B) [36,63]. In Δ-s-tether and *osh4*Δ Δ-s-tether cells, however, multicopy *DGK1* induced a slight but statistically significant increase in ER in close vicinity to the PM. *DGK1* overexpression still does not restore extensive cortical ER in Δ-s-tether and *osh4*Δ Δ-s-tether cells but limited reestablishment of ER-PM association, even without directed tethering, might suppress *osh4*Δ Δ-s-tether lethality.

Multicopy *DGK1* also rectified *INO1* dysregulation by changing the relative expression of the key activators and repressors controlling phospholipid gene transcription. In *osh4-1*^ts^ Δ-s-tether cells at 37°C for 1 h, *OPI1* repressor expression is reduced ~3 fold with or without the *DGK1* suppressor (Fig 13C). As predicted, reduced *OPI1* contributes to an increase of *INO1* mRNA. In *osh4-1*^ts^ Δ-s-tether cells at 37°C, the transcriptional activator *INO2* is inhibited ~4 fold, which is predicted to reduce *INO1* transcription. With multicopy *DGK1*, *INO1* expression is nearly restored to WT levels. In *DGK1* suppressed *osh4-1* Δ-s-tether cells, 89% of lipid biosynthesis (40/45) and 78% of inositol-regulated genes (97/124) are normally expressed. This finding suggests that the downregulation of *OPI1* repressor mRNA is offset by further inhibition of *INO2* activator expression. *DGK1* suppression appears to rescue the normal regulatory balance of phospholipid gene transcription, which might contribute to membrane stress mitigation in *osh4-1*^ts^ Δ-s-tether cells. Together by partial restoration of ER-PM association and resetting the balance of the regulators of phospholipid gene transcription, *DGK1* suppression appears to moderate ESR-related defects caused by the absence of ER-PM MCSs [3].

## Discussion

ER-PM MCSs serve as a regulatory nexus for coordinating lipid biosynthesis and as likely zones for lipid transfer between the ER and PM [3,8,33,64,65]. As presumptive components of many MCSs, it is counterintuitive that eliminating yeast ORPs does not reduce ER-PM MCSs but rather leads to their proliferation. However, we found that *OSH* gene elimination led to the compensatory generation of extra Tcb3p-dependent ER-PM MCSs. Although Tcb3p induction is post-transcriptional, clustering analysis of the transcriptomic data indicated that *OSH* gene inactivation elicits the global ESR pathway, which counteracts cytotoxic stress. It is interesting that *OSH* gene inactivation, which increases ER-PM MCSs, and the deletion of ER-PM tether genes, which decrease ER-PM MCSs, both elicit the ESR pathway. This finding suggests that ER-PM MCS homeostasis is closely monitored by the ESR pathway. *OSH* gene deletion triggers iESR changes through responses to PM stress involving the HOG pathway. In contrast, ER stress and UPR activation contributed to iESR changes in cells lacking ER-PM tether genes. Consistent with these findings, disruption of HOG or UPR pathways also increases Tcb3p-containing ER-PM MCSs leading to more cortical ER association with the PM. As such, ER-PM MCSs represent stabilizing factors to membrane stresses that disrupt either the ER or PM.

Among all ER-PM tethers, *TCB1*-*TCB3* have been shown to play a key role in maintaining PM integrity in response to heat stress [66]. In addition, *TCB3* specifically confers tolerance to chronic ER stress [67]. We show that in response to these stresses, Tcb3p-dependent ER-PM tethering increases but the mechanism is relatively general given that increasing ER-PM MCSs with the non-specific artificial membrane staple also protects against ER stress. The ER-PM staple can bypass the requirement of UPR signaling when cells are treated with otherwise lethal ER stress. This rescue was accomplished without a general expansion of ER, which suggests a different mechanism than previously reported [36]. Regardless, attaching bilayers together during stress might provide a structural buttressing in which both membranes are physically molded and reinforced by their overlay [10]. Alternatively, increased ER-PM MCSs might confer lipid regulatory changes to maintain membrane integrity by modulating lipid composition [6,10,19]. Indeed, lipid bilayer stress confers a specific UPR program independent of proteotoxicity [53], and disrupting *OSH* and ER-PM tether function disrupts phospholipid metabolism and results in Opi1p-dependent phospholipid dysregulation [3,21]. Of course, these mechanisms are not mutually exclusive.

Osh protein elimination increased the post-translational expression of Tcb3p, despite that *TCB1-TCB3* and *IST2* transcription is reduced 2-3-fold in *osh4-1*^*ts*^ *osh*Δ cells. Although gene and protein expression do not always directly correlate, negative feedback loops are common modes to reassert homeostatic control of membrane maintenance [68]. Another possibility is that rESR-mediated global transcriptional decreases tether gene expression, which in *osh4-1*^*ts*^ *osh*Δ cells is offset by increased stability of Tcb3p in ER-MCSs. Thus, as a membrane protective response, the stabilization of Tcb3p in ER-PM MCSs might facilitate recovery after severe ER and PM perturbation.

Consistent with previous reports [2], the RNA-seq profile of Δ-s-tether cells indicated a constitutive activation of UPR signaling, though we found that this response was attenuated by inositol. Although *OSH3* is a UPR induced gene [69], *OSH3* is not induced in Δ-s-tether cells (only *OSH5* and *OSH6* showed any transcriptional increases, and *OSH1* expression is repressed) and the combined inactivation of all *OSH* genes had a minor impact on UPR gene expression (Figs 4 and 5). However, the transcriptomic profile of cells in which *osh4-1*^*ts*^ and Δ-s-tether mutations are combined, also combines the associated UPR and heat shock/PM stress responses, which are integrated through the ESR. These membrane stresses are further compounded by phospholipid dysregulation that is in part due to Opi1p mislocalization caused by *OSH* or ER-PM tether deletions.

*INO1* expression is significantly induced in Δ-s-tether and *osh4-1*^*ts*^ Δ-s-tether cells, though the increase is greatest in *osh4-1*^*ts*^ *osh*Δ cells. Due to the defects in nuclear translocation of Opi1p, inositol-dependent repression of *INO1* transcription is defective in Δ-s-tether and *osh4-1*^*ts*^ *osh*Δ cells, albeit for different reasons. In Δ-s-tether cells, Opi1p is mistargeted to a completely different organelle away from the nucleus, whereas the absence of *OSH*s causes the localization of Opi1p to stay on the ER. These results explained the expression defects in phospholipid gene regulation that likely contributed to the membrane stresses leading to ESR gene changes.

Despite that the genetic selection was saturated for strong suppressors of *osh4*Δ Δ-s-tether lethality, no novel tether factor was isolated. *DGK1* was the only strong extragenic suppressor isolated and was independently isolated 12 times. Dgk1p has no clear attributes of a known tether protein and does not directly contribute to ER-PM tethering. However, there are technical limitations to dosage suppression selections that might prevent the isolation of some suppressor genes. For instance, relevant genes would not be identified if: (i) they are toxic when overexpressed; (ii) they are larger than the size selection for the genomic library (i.e. < 10 kb); (iii) they are part of a multi-subunit complex that is contingent on several genes for functionality. In addition, a survey of upregulated genes in the *osh4-1* Δ-s-tether transcriptome did not identify any known ER or PM associated factors that might encode other inducible tethers (*VPS13* and *SIP3* are both upregulated, but neither rescue *osh4-1* Δ-s-tether lethality when overexpressed). Based on our findings, we suggest three possibilities for the mode of *DGK1* bypass suppression of *osh4*Δ Δ-s-lethality. First, in the absence of the tethers deleted in the Δ-s-tether cells, *DGK1* overexpression induces a novel ER-PM tether. In other cellular and environmental contexts additional tether proteins undoubtedly provide significant ER-PM association to foster specific physiological or metabolic functions. Such factors were not identified through the dosage suppression experiment, but again this approach would not isolate unusually large genes or those involved in multi-subunit ER tethers, which without the other components might lack the intrinsic ability to mediate PM contact (e.g. Scs2p, Scs22p and Ice2p). Second, in our analysis of the *DGK1* bypass suppression of *osh4Δ* Δ-s-tether lethality, a minor amount of cortical ER was restored by multicopy *DGK1* in Δ-s-tether strains (Fig 13A and 13B). This result was a surprise considering that in WT cells cytoplasmic ER expansion around the nucleus is induced without affecting cortical ER (Fig 13A and 13B) [36,63]. It is therefore possible that this minimal amount of cortical ER might be enough to rescue *osh4Δ* Δ-s-tether growth. In this scenario, the general expansion of amassed ER by *DGK1* overexpression, as opposed to the directed attachment of ER to the PM, might indirectly restore small amounts of ER-PM association. Third, Dgk1p/diacylglycerol kinase overexpression increases phospholipid synthesis flux in WT cells [63]. By generating greater lipid flux for phospholipid biosynthesis, the mechanism of *DGK1* bypass suppression might correct phospholipid metabolic defects as detected in ER-PM tether mutants [3,6]. Indeed, increased diacylglycerol kinase activity is predicated to alleviate the observed accumulation of DAG in Δ-s-tether cells, thereby restoring normal flux through the phospholipid biosynthetic pathway. Given that Osh proteins control membrane organization and dynamics [21], *DGK1* restoration of phospholipid flux might also suppress contributing defects associated with *OSH4* inactivation in Δ-s-tether cells. Future studies will focus on discerning between these possibilities.

As important determinants of membrane function, ORPs and the Osh family of proteins have various proposed activities in vesicular and non-vesicular transport involving many different internal membranes [17,18,33,51,70]. Through their FFAT motifs, the long Osh proteins (Osh1p–Osh3p) interact with the membrane tether Scs2p, which is an integral ER membrane protein that has no domain able to bind the PM by itself [12,71]. As potential bridging subunits, the removal of long Osh proteins would be predicted to decrease ER-PM association, like that observed in *scs2*Δ cells. However, like the inactivation of all Osh proteins, deletion of just the long Osh proteins induces ER-PM MCS proliferation and cortical ER-PM association. This finding suggests that the long Osh proteins are specifically relevant to ER-PM MCS regulation, despite the synthetic genetic interaction between *OSH4* and Δ-s-tether deletions. Indeed, Osh2p interferes with endocytic internalization and has been suggested to act as negative regulator of ER-PM MCSs and cortical ER [72]. Regardless, the Tcb3p-dependent compensatory mechanism that increases ER-PM junctions in the absence of Osh proteins appears to be unique when considering other ORP-dependent membrane contacts. Membrane contact between the vacuole-nuclear ER contact requires Osh1p to directly mediate physical contact without any compensatory mechanism [48,49]. The role of Osh proteins in ER-lipid droplet contact appears to be more complicated. When Osh proteins are eliminated the overall proportion of lipid droplets contacting the nucleus does not change relative to WT, even though there are much more lipid droplets. Osh proteins appear to have more of an effect on lipid droplet biogenesis than contact with other organelle membranes. Depending on membrane context, Osh proteins have distinct functional roles depending on which membrane contact site is involved.

## Materials and methods

### Strains, plasmids, microbial techniques

Yeast strains and plasmids are listed in S1 Table. Unless otherwise stated, yeast cultures were grown in synthetic complete, synthetic defined or YPD rich media at 30°C. To test growth defects, the *osh4-1*^*ts*^ *osh*Δ or *osh4-1*^*ts*^ Δ-s-tether temperature conditional mutants were cultured to mid-log phase at 30°C, resuspended in pre-warmed media at 37°C, and then incubated at 37°C for 1 h. DNA cloning, bacterial and yeast transformations were conducted using standard techniques [73,74]. All images shown of yeast cultures on solid growth media are representative of at least three independent trials.

To generate functional GFP-*IST2* and GFP-*SCS2* integrations, in which the fusions are under transcriptional control of their native promoters, *sf*GFP-*IST2* and -*SCS2* were PCR amplified from WKY0164 and WKY0133 [14]. From the amplified *sf*GFP-*SCS2* sequence, a 2 kb NotI/NsiI *sf*GFP-*SCS2* fragment and a KpnI/NotI 0.8 kb 3’ *SCS2* fragment were ligated and subcloned into the NsiI/NotI sites of YIplac211 to create the pCB1418 GFP-*SCS2* integration construct. A 4.4 kb NotI/SphI *sf*GFP-*IST2* fragment and a NsiI/NotI 0.9 kb 3’ *IST2* sequence were ligated into the NsiI/SphI sites of YIplac211 to create the pCB1417 GFP-*IST2* integration construct. Integrated constructs in yeast were confirmed by PCR and functionally verified as correct by proper GFP fusion localization.

To test choline sensitivity, cells were grown on solid synthetic complete media with and without 1 mM choline chloride (Sigma-Aldrich Chemicals) and incubated at 30°C. Growth in response to inositol supplementation was tested on solid synthetic minimal media containing 75 μM myo-inositol (Sigma-Aldrich Chemicals). To examine localization of Opi1-GFP by confocal fluorescence microscopy, cells were cultured in liquid synthetic media with the addition of 300 μM inositol for 1 h. For UPR activation, cells were treated in liquid media with 2 mM dithiothreitol (DTT) for 2 h or cultured on solid synthetic minimal media containing 4 mM DTT (Sigma-Aldrich Chemicals). Growth in response to osmotic stress was tested on solid and liquid growth media containing 0.7 M sodium chloride (Sigma-Aldrich Chemicals) or 1 M sorbitol (BioShop Canada Inc, Burlington, Ontario). To select against *URA3*-marked plasmids (e.g. pSCS2), yeast cultures were grown on rich growth media and then transferred onto synthetic solid growth medium containing 1 g/L 5’-fluoroorotic acid (Gold Biotechnology, St. Louis, MO). To select against *TRP1*-marked plasmids, yeast cultures were grown on selective growth media and then transferred onto a synthetic solid medium containing 0.5 g/L 5-fluoroanthranilic acid (5-FAA; Toronto Research Chemicals, Ontario, CA) supplemented with tryptophan to a final concentration of 200 mg/l. To test *ire1*Δ and *hog1*Δ lethality in *osh1*Δ *osh2*Δ *osh3*Δ cells, *IRE1* and *HOG1* were deleted in the *osh1*Δ *osh2*Δ *osh3*Δ strain (JRY62563) carrying a *TRP1*-marked plasmid copy of *OSH2* (pCB113). With the *OSH2* plasmid the *hog1*Δ *osh1*Δ *osh2*Δ *osh3*Δ (CBY6914) and *ire1*Δ *osh1*Δ *osh2*Δ *osh3*Δ (CBY6916) cells grow, but after 5-FAA treatment these strains are inviable without benefit of the counter-selected *OSH2* plasmid.

### Electron microscopy

For cryofixation and electron microscopy of conditional mutants, yeast cells were grown to mid-log phase at 30°C and then cultured at 37°C as described above. High-pressure freezing, freeze substitution, and sample preparation were performed as previously described [75]. High-pressure freezing was performed using a Leica HPM100 High Pressure Freezer (Vienna, Austria). Cells were dehydrated and stained using 2% osmium tetroxide plus 0.1% uranyl acetate in acetone using a Leica AFS2 automatic freeze substitution system (Vienna, Austria). Cell samples were then rinsed in 100% acetone three times at room temperature and gradually infiltrated with an equal part mixture of Spurr’s and Gembed embedding media. 50 nm thin sections were prepared using a Diatome ultra 45° knife (Diatome, Switzerland) and transmission electron microscopy was performed on a Hitachi H7600 TEM (Hitachi HighTech, Tokyo, Japan) at 80.0kV. Determinations of cortical ER abundance in electron micrographs, and the ratio of plasma membrane associated with cortical ER, were made using ImageJ (https://imagej.nih.gov/ij/). Cortical ER was assigned as previously described [76].

### Fluorescence microscopy

Confocal fluorescence microscopy was performed as previously described [77]. Super-resolution fluorescence microscopy was performed on a Zeiss LSM 880 Confocal microscope with an Airyscan super-resolution GaAsP detector and 63x/1.4 oil immersion objective (Zeiss, Oberkochen, Germany). All fluorophores were acquired using pixel dwell times at 1.31 μs per pixel. GFP-Opi1p, Tcb3p-GFP and DsRed-HDEL were excited using a 488 nm and 561 nm lasers, respectively. Relative laser intensities for 488 nm laser and 561 nm laser were both set to 1.5 and the digital gain was set to 900. Images were Airyscan processed in Zen Black and deconvolved in Zen Blue using fast-iterative deconvolution (Zeiss Canada, North York, ON, Canada). Cortical association was assayed by tracing the cell cortex in the Zen Blue profile mode, then measuring cortical fluorescence intensity at the cortex. Cortical association was expressed as a ratio of the total distance of cortical fluorescence to the total cortical distance. Images were exported as 8-bit uncompressed TIFF files then processed in Affinity Photo (Serif Ltd., Nottingham, UK).

### Immunoblot analysis

For analysis of Tcb3, Ist2 and Scs2 protein expression, cells were grown to mid log-phase at 30°C or temperature conditional mutants were shifted to the restrictive growth temperature as described above. 10 OD_600_ units of Tcb3p-GFP, GFP-Ist2p or -Scs2p expressing cells were prepared as previously described [78]. For GFP-Scs2p immunoblots, pellets were resuspended in SDS sample buffer and heated for 10 min at 50°C before electrophoresis. For Tcb3p-GFP and GFP-Ist2p, pellets were resuspended in SDS sample buffer containing 8 M urea, and then heated for 10 min at 50°C. Protein transfer to nitrocellulose membranes and immunoblot conditions were as previously described [79]. To detect Tcb3p-GFP, GFP-Ist2p or -Scs2p, immunoblots were incubated with a 1:1000 anti-GFP antibody (ThermoFisher Scientific Inc., Waltham, MA) followed with 1:10,000 anti-rabbit-HRP secondary antibody (Bio-Rad Laboratories, Mississauga, ON). Actin was detected using 1:1000 anti-actin antibody (Cedarlane, Burlington, ON) followed with 1:10000 anti-mouse-HRP secondary antibody (ThermoFisher Scientific Inc.).

### Genomic expression analysis

For transcriptome analysis of s-tether cells, WT and Δ-s-tether cells were grown to mid-log phase at 30°C in synthetic minimal media with and without 75 μM inositol as stated. For transcriptome analysis of all temperature sensitive cells, WT, *osh4-1*, *osh4-1*^ts^ *osh*Δ cells, *osh4-1*^*ts*^ Δ-s-tether and *osh4-1*^*ts*^ Δ-s-tether [*DGK1*] cells were grown to mid-log phase at 30°C in synthetic minimal media then shifted to 37°C for 1 h. To test the effect of inositol supplementation, WT, *osh4-1 osh*Δ, and Δ-s-tether cells were cultured in synthetic minimal media containing 75 μM inositol. A total of 2 OD_600_ units were pelleted and frozen in liquid nitrogen for RNA-seq analysis. RNA was isolated using mechanical lysis using acid-washed glass beads, and the Monarch Total RNA Miniprep Kit (NEB). Samples were poly(A) enriched with NEBNext poly(A) mRNA magnetic Isolation beads (NEB) using 1 μg of total RNA input. cDNA libraries were generated using NEBNext Ultra II RNA Library kit (NEB). Library quality was analyzed using an Agilent-Bioanalyzer 2100. Sequencing was performed on an Illumina NextSeq500 (Illumina, San Diego, CA) except for Δ-s-tether cell samples (cultured in synthetic minimal media at 30°C), which were sequenced on an Illumina MiSeq (Illumina). A total of three biological replicates were performed for each condition except for *osh4-1* and *osh4-1* Δ-s-tether [*DGK1*], which were performed in duplicate. All samples generated >1.0 x 10^7^ paired-end reads except Δ-s-tether cells in synthetic minimal medium, which generated a total of 4.3 x 10^6^ ± 0.7 x 10^6^ paired-end reads.

All read quality control, alignment, read counting, and differential expression analysis was performed using Illumia Base space. The reads were aligned to *Saccharomyces cerevisiae* Ensembl release 61 genome and counted using RNAstar [80]. Differential expression analysis was performed using DESeq2 (v2.11.40.2) [81]. After differential expression analysis, ORFs corresponding to auxotrophic markers, mating types, tRNA, snRNA, dubious open reading frames, TY elements, *OSH* and/or tether genes were removed from subsequent analyses; a total of 1608 ORFs were removed. Gene ontology and statistical analysis were performed in RStudio. KEGG analysis was performed using the enrichKEGG function of clusterProlifer (v3.14) [82] and Metascape [83]. Scatter plots and paired correlation analysis were performed using ggplot2 [84], and heatmap analyses was performed using heatmap.2 in the gplot R package [85]. By combining high-throughput and manually curated gene transcriptional profiles of known *HOG1* downstream transcription factors as listed the Saccharomyces Genome Database (SGD), *HOG* pathway transcription targets were compiled. Similarly, heat shock pathway transcription targets were manually curated from SGD by combining transcriptional targets of heat shock transcription factor 1 (Hsf1p), and UAS_ino_ target genes represent transcriptional targets of Ino2p and Ino4p. UPR target genes were curated as previously reported [86]. ESR gene lists were compiled from previous reports [51,87].

For qPCR validation of RNA-seq data, mRNA from WT and *osh4-1*^*ts*^ Δ-s-tether cells was extracted as described above and cDNA was synthesized using LunaScript RT Supermix Kit (New England Biolabs, MA). 5 μL of RNA (1 μg), 4 μL of 5x LunaScript RT SuperMix and 11 μL of nuclease-free water were mixed for cDNA synthesis in a thermal cycler using the program: 25°C for 2 min, 55°C for 10 min and 95°C for 1 min for 1 cycle. *GRE2*, *KAR2* and *SIP18* genes were amplified from diluted (1:200) cDNA, and amplified *ACT1* was the internal qPCR control. For each target gene, primers were generated using the Primerquest Tool (www.idtdna.com/pages/tools/primerquest) and 2 primer pairs capable of amplifying DNA fragments of ~200 bp were chosen. 8 μL of diluted cDNA, 1 μL each of forward and reverse primers (5 μM), and 10 μL of Luna Universal qPCR Master Mix were added to wells of a qPCR plate sealed with an optically transparent film. RT-qPCR of each gene was performed in triplicate in an Applied Biosystems Quantstudio 3 Real-Time PCR system using SYBR scan mode. The thermal cycling program used was: 1 min at 95°C, 40 cycles of 95°C for 15 s and 60°C for 1 min, and heat inactivation for 1 min at 95°C. Melting curve analysis was performed using the default program of the QuantStudio 3 qPCR machine.

### Extragenic suppressor selection

To isolate extragenic suppressors of *osh4-1*^*ts*^ Δ-s-tether synthetic lethality, we used a 2μ high-copy genomic plasmid library. SEY6210 genomic DNA was partially digested using Sau3AI and 10 kb fragments were size selected from an agarose gel and ligated into BamHI digested YEplac195. This 2μ genomic library was then transformed into *osh4-1*^*ts*^ Δ-s-tether cells and cultured on solid synthetic complete medium lacking leucine and uracil for 3–7 days at 37°C. Suppressor colonies from an equivalent of 7500 transformants (~5 genomic equivalents) were selected. Viable suppressor colonies at 37°C were colony purified and confirmed after recovery and re-transformation of genomic library plasmids back into *osh4-1*^*ts*^ Δ-s-tether cells to affirm suppression at 37°C. To identify individual suppressing genes from within the several genes on each genomic plasmid fragment, candidate genes were individually cloned into YEplac195 and tested for suppression at 37°C after transformation into *osh4-1*^*ts*^ Δ-s-tether cells.

## Supporting information

S1 Table **A**. Yeast strains, and **B**. Plasmids.
(DOCX)

S1 Fig *OSH2* deletion in Δ-s-tether cells does not impact growth. WT (SEY6210), *osh4Δ* Δ-s-tether (CBY5988) and *osh2Δ* Δ-s-tether (CBY6734) cells that contain *SCS2* on a *URA3*-marked plasmid (pSCS2) were streaked onto solid growth medium. Cells were cultured for 4 days at 30°C on growth medium (containing 5’-FOA) to select against the *SCS2*-containing plasmid (-*SCS2*). As compared to growth on standard synthetic medium (+*SCS2*), *osh4Δ* Δ-s-tether cells do not growth in the absence of *SCS2*, whereas growth of *osh2*Δ Δ-s-tether cells is not dependent on *SCS2*.
(TIF)

S2 Fig KEGG pathway and heatmap analysis of *osh4-1*^ts^ Δ-s-tether, Δ-s-tether, and *osh4-1*^*ts*^ *osh*Δ genomic expression. (**A)** Gene expression relative to WT (SEY6210) in *osh4-1*^ts^ Δ-s-tether (CBY6031), Δ-s-tether (CBY5838), and *osh4-1*^*ts*^ *osh*Δ (CBY926) cells listed by KEGG category. (**B)** Relative to WT, heatmap analyses of transcriptional responses in *osh4-1*^*ts*^ Δ-s-tether, Δ-s-tether, and *osh4-1*^*ts*^ *oshΔ* cells affecting inositol metabolism and regulation, lipid metabolism, and ERAD gene expression. Blue bars indicate transcriptional induction and red indicates repression.
(TIFF)

S3 Fig qPCR analysis of *osh4-1*^ts^ Δ-s-tether gene expression. Duplicate analyses of *GRE2*, *KAR2*, and *SIP18* transcript levels by RT-qPCR in WT (SEY6210) and *osh4-1*^ts^ Δ-s-tether (CBY6031) cells at 37°C for 1 h. Each analysis represents three measurements, and error bars indicate SEM.
(TIF)

S4 Fig iESR and rESR gene expression in Δ-s-tether, *osh4-1*, *osh4-1*^ts^ *oshΔ*, *and osh4-1*^ts^ Δ-s-tether cells. Heatmap analyses of transcriptional responses relative to WT (SEY6210) at 37°C for 1 h in *osh4-1* (CBY7177), *osh4-1*^*ts*^ Δ-s-tether (CBY6031), Δ-s-tether (CBY5898), and *osh4-1*^*ts*^ *oshΔ* (CBY926) cells affecting **(A)** iESR and (**B)** rESR genes. Downregulated genes are shown in red, upregulated genes are shown in blue. iESR and rESR regulated genes were curated from previous reports [52,87].
(TIFF)

S5 Fig Transcriptional anticorrelation of specific gene groups in Δ-s-tether versus *osh4-1*^ts^ *osh* cells. Anticorrelation of transcriptional responses in Δ-s-tether (CBY5898) versus *osh4-1*^*ts*^ *oshΔ* (CBY926) cells. Reciprocal expression of specific GO-term gene groups are shown either when reduced in *osh4-1*^*ts*^ *oshΔ* cells but increased in Δ-s-tether cells, or increased in *osh4-1*^*ts*^ *oshΔ* cells and decreased in Δ-s-tether cells.
(TIFF)

S6 Fig Inositol effects on Δ-s-tether and *OSH* mutant cells. (**A)** Scatter plot analysis of gene expression in Δ-s-tether (CBY5838) cells, relative to WT (SEY6210), when cultured in the absence versus presence of exogenously added inositol. (**B)** Scatter plot analysis of *osh4-1*^*ts*^ *osh*Δ (CBY926) gene expression, relative to WT, when cultured in the absence versus presence of exogenously added inositol. (**C)** Ten-fold serial dilutions of WT, Δ-s-tether, *osh4-1*^*ts*^ *osh*Δ, *osh1Δ osh2Δ osh3*Δ (JRY6253), and *osh4Δ osh5Δ osh6*Δ *osh7Δ* (JRY6272) cells grown at 30 or 37°C on solid synthetic minimal media containing 75 μM myo-inositol, 1 mM choline, or 75 μM myo-inositol with 1 mM choline.
(TIFF)

S7 Fig Expression of *OSH*, tether, and high-copy suppressors genes in Δ-s-tether, *osh4-1*^ts^ *oshΔ*, *and osh4-1*^ts^ Δ-s-tether cells. **(A)** Transcriptional expression of *OSH1-OSH7* in Δ-s-tether (CBY5838) cells relative to WT (SEY6210) cells cultured with or without 75 μM inositol. (B) Transcriptional expression of genes encoding primary tether proteins in *osh4-1*^*ts*^ *osh*Δ (CBY926) cells relative to WT cells cultured with or without inositol. (C) *DGK1* expression in *osh4-1*^*ts*^ *osh*Δ, Δ-s-tether, and *osh4-1*^ts^ Δ-s-tether (CBY6031) cells relative to WT. (D) Transcriptional expression of ER-PM tether genes in *osh4-1* (CBY7177) and *osh4-1*^*ts*^ *osh*Δ cells relative to WT.
(TIFF)

S8 Fig Correlative analysis of *osh4-1*^*ts*^ Δ-s-tether, Δ-s-tether, and *osh4-1*^*ts*^ *osh*Δ transcriptomic profiles. Correlation matrix of relative transcript abundance in *osh4-1*^*ts*^ Δ-s-tether (CBY6031), Δ-s-tether (CBY5898), and *osh4-1*^*ts*^ *oshΔ* (CBY926) cells relative to WT (SEY6210) grown in synthetic minimal medium at 30°C; *osh4-1*^*ts*^ Δ-s-tether and *osh4-1*^*ts*^ *oshΔ* cells were then incubated at 37°C for 1 h, as was their comparative WT control. Pearson correlations of corresponding genotypes as shown.
(TIF)

S9 Fig *IRE1* and *HOG1* stress responses are independent under the conditions used in this study. Ten-fold serial dilutions of WT, *ire1Δ*, and *hog1Δ* cells grown at 30°C on solid synthetic minimal media with and without 0.7 M NaCl or 4 mM DTT. Cells lacking *IRE1* are sensitive to DTT but not to NaCl treatment and, inversely, cells lacking *HOG1* are sensitive to NaCl treatment but not DTT treatment.
(TIFF)

S10 Fig Multicopy *OSH* gene suppressors of *osh4-1*^ts^ Δ-s-tether. Tenfold serial dilutions of WT (SEY6210), Δ-s-tether (CBY5898), and *osh4-1*^*ts*^ Δ-s-tether (CBY6031) transformed with the 2μ plasmid control (YEplac195), *OSH1* (pCB240), *OSH2* (pCB239), *OSH3* (pCB238), *OSH4* (pCB241), *OSH5* (pCB242), *OSH6* (pCB237), or *OSH7* (pCB236). Cells were grown on synthetic complete medium for 3–5 days at 23 or 37°C
(TIFF)

S1 File Raw data. (XLSX)
