# Supplementary material for: ER-PM membrane contact site regulation by yeast ORPs and membrane stress pathways
Source: PLoS Genet. 2022 Mar 3;18(3):e1010106. doi: 10.1371/journal.pgen.1010106 (PMC8923467; doi:10.1371/journal.pgen.1010106)
Supplement: S1 Table — A. Yeast strains, and B. Plasmids. (DOCX) [file pgen.1010106.s001.docx]

**Supporting information**

**S1 Table A. Yeast Strains.**

| **Strain** | **Genotype** | **Reference** |
| --- | --- | --- |
| BY4741 | *MAT***a** *leu2Δ0 ura3Δ0 his3Δ0 met15Δ0* | [1] |
| BY4742 | *MAT*𝛼 *leu2Δ0 ura3Δ0 lys2Δ0 his3Δ0* | [1] |
| CBY924 | SEY6210 *osh1∆*::*kanMX4 osh2∆*::*kanMX4 osh3∆*::*LYS2 osh4∆*::*HIS3 osh5∆*::*LEU2 osh6∆*::*LEU2 osh7∆*::*HIS3* [*OSH4 TRP1 CEN*] | [2] |
| CBY926 | SEY6210 *osh1∆*::*kanMX4 osh2∆*::*kanMX4 osh3∆*::*LYS2 osh4∆*::*HIS3 osh5∆*::*LEU2 osh6∆*::*LEU2 osh7∆*::*HIS3* [*osh4-1 TRP1 CEN*] | [2] |
| CBY1048 | BY4741 *ire1∆*::*kanMX4* | [1] |
| CBY5838 | SEY6210 *ist2Δ*::*hisMX6 scs2Δ*::*TRP1 scs22Δ*::*hisMX6 tcb1Δ*::*kanMX6 tcb2Δ*::*kanMX6 tcb3Δ*::*hisMX6 ice2Δ*::*natMX4* | [3] |
| CBY5988 | SEY6210 *ist2Δ*::*hisMX6 scs2Δ*::*TRP1 scs22Δ*::*hisMX6 tcb1Δ*::*kanMX6 tcb2Δ*::*kanMX6 tcb3Δ*::*hisMX6 osh4∆*::*hphMX4 ice2∆*::*natMX4* [*SCS2 URA3 CEN*] | [3] |
| CBY6031 | SEY6210 *ist2Δ*::*hisMX6 scs2Δ*::*TRP1 scs22Δ*::*hisMX6 tcb1Δ*::*kanMX6 tcb2Δ*::*kanMX6 tcb3Δ*::*hisMX6 osh4∆*::*hphMX4 ice2∆*::*natMX4* [*osh4-1 LEU2 CEN*] | [3] |
| CBY6087 | SEY6210 *TCB3-*GFP:*URA3* |  |
| CBY6091 | CBY926 *TCB3-*GFP:*URA3* |  |
| CBY6465 | BY4741 *hog1∆*::*kanMX4* | [1] |
| CBY6506 | SEY6210 *ist2Δ*::*hisMX6 scs2Δ*::*TRP1 scs22Δ*::*hisMX6 tcb1Δ*::*kanMX6 tcb2Δ*::*kanMX6 tcb3Δ*::*hisMX6 ice2Δ*::*natMX4 osh4*∆:: *hphMX4* [*DGK1 LEU2* 2μ] |  |
| CBY6912 | SEY6210 *hog1∆*::*kanMX4* [*OSH2 TRP1* 2μ] |  |
| CBY6914 | SEY6210 *osh1∆*::*URA3 osh2∆*::*URA3 osh3∆*::*LYS2 hog1∆*::*kanMX4* [*OSH2 TRP1* 2μ] |  |
| CBY6916 | SEY6210 *osh1∆*::*URA3 osh2∆*::*URA3 osh3∆*::*LYS2 ire1∆*::*kanMX4* [*OSH2 TRP1* 2μ] |  |
| CBY6734 | SEY6210 *ist2Δ*::*hisMX6 scs2Δ*::*TRP1 scs22Δ*::*hisMX6 tcb1Δ*::*kanMX6 tcb2Δ*::*kanMX6 tcb3Δ*::*hisMX6 osh2∆*::*hphMX4 ice2∆*::*natMX4* [*SCS2 URA3 CEN*] |  |
| CBY7177 | SEY6210 *osh4*∆::*HIS3* [*osh4-1 LEU2 CEN*] |  |
| CBY7272 | SEY6210 *TCB3-*GFP:*HIS3* |  |
| CBY7274 | SEY6210 *osh1∆*::*URA3 osh2∆*::*URA3 osh3∆*::*LYS2 TCB3-*GFP:*HIS3* |  |
| CBY7298 | SEY6210 *osh4*∆::*HIS3 osh5*∆::*LEU2 osh6*∆::*LEU2 osh7*∆::*HIS3 TCB3-*GFP:*URA3* |  |
| CBY7300 | SEY6210 *sf*GFP -*IST2*:*URA3* |  |
| CBY7302 | CBY926 *sf*GFP-*IST2*:*URA3* |  |
| CBY7304 | SEY6210 *sf*GFP-*SCS2*:*URA3* |  |
| CBY7306 | CBY926 *sf*GFP-*SCS2*:*URA3* |  |
| CBY7354 | BY4742 *tcb3*∆::*kan*MX4 | [1] |
| CBY7392 | SEY6210 *osh1∆*::*URA3 osh2∆*::*URA3 osh3∆*::*LYS2 tcb3*∆::*kan*MX4 |  |
| CBY7398 | SEY6210 *tcb3*∆::*kan*MX4 |  |
| JRY6253 | SEY6210 *osh1∆*::*URA3 osh2∆*::*URA3 osh3∆*::*LYS2* | [4] |
| JRY6272 | SEY6210 *osh4∆*::*HIS3 osh5∆*::*LEU2 osh6∆*::*LEU2 osh7∆*::*HIS3* | [4] |
| SEY6210 | *MAT*𝛼 *leu2‐3,112 ura3‐52 his3Δ200 trp1Δ901 lys2‐801 suc2Δ9* | [5] |
| WKY0133 | *MAT***a** *leu2-3,112*::*GAL1pr-I-SCEI-natNT2 ura3-52 his3Δ200 lys2-801 sfGFP-SCS2* | [6] |
| WKY0164 | *MAT***a** *leu2-3,112*::*GAL1pr-I-SCEI-natNT2 ura3-52 his3Δ200 lys2-801 sfGFP-IST2* | [6] |

Unless otherwise stated, all strains were created as part of this study.

**S1 Table B. Plasmids.**

| **Plasmid** | **Description** | **Reference** |
| --- | --- | --- |
| pCB113 | *OSH2 TRP1* 2μ |  |
| pCB236 | *OSH7 URA3* 2μ | [7] |
| pCB237 | *OSH6 URA3* 2μ | [7] |
| pCB238 | *OSH3 URA3* 2μ | [7] |
| pCB239 | *OSH2 URA3* 2μ | [7] |
| pCB240 | *OSH1 URA3* 2μ | [7] |
| pCB241 | *OSH4 URA3* 2μ | [7] |
| pCB242 | *OSH5 URA3* 2μ | [7] |
| pCB1157 | *osh4-1^ts^ LEU2 CEN* | [3] |
| pCB1185 | P*^ACT1^*-GFP-Myc*-HMH-RitC LEU2 CEN* | [3] |
| pCB1346 | *DGK1* *LEU2* 2μ |  |
| pCB1417 | *sf*GFP-*IST2 URA3* |  |
| pCB1418 | *sf*GFP-*SCS2 URA3* |  |
| pRS416-DsRED-HDEL | DsRed-HDEL *URA3* *CEN* | [8] |
| pRS415-DsRED-HDEL | DsRed-HDEL *LEU2* *CEN* | Gift from Scott Emr |
| pRS416-PHO5-GFP-Opi1 | P*^PHO5^*-GFP-*OPI1* *URA3* *CEN* | Gift from Chris Loewen |
| pSCS2 | P*^PHO5^*-Myc-*SCS2 URA3 CEN* | [9] |
| YCplac111 | *LEU2 CEN* | [10] |
| YEplac181 | *LEU2* 2μ | [10] |
| YEplac195 | *URA3* 2μ | [10] |
| YIplac211 | *URA3* | [10] |

Unless otherwise stated, all plasmids were created as part of this study.

**References:**

1. Winzeler EA, Shoemaker DD, Astromoff A, Liang H, Anderson K, Andre B, et al. Functional characterization of the S. cerevisiae genome by gene deletion and parallel analysis. Science 1999;285(5429):901-6. pmid:10436161.

2. Beh CT, Rine J. A role for yeast oxysterol-binding protein homologs in endocytosis and in the maintenance of intracellular sterol-lipid distribution. J Cell Sci 2004;117(Pt 14):2983-96. pmid:15173322.

3. Quon E, Sere YY, Chauhan N, Johansen J, Sullivan DP, Dittman JS, et al. Endoplasmic reticulum-plasma membrane contact sites integrate sterol and phospholipid regulation. PLoS Biol 2018;16(5):e2003864. pmid:29782498.

4. Beh CT, Cool L, Phillips J, Rine J. Overlapping functions of the yeast oxysterol-binding protein homologues. Genetics 2001;157(3):1117-40. pmid:11238399.

5. Robinson JS, Klionsky DJ, Banta LM, Emr SD. Protein sorting in Saccharomyces cerevisiae: isolation of mutants defective in the delivery and processing of multiple vacuolar hydrolases. Mol Cell Biol 1988;8(11):4936-48. pmid:3062374.

6. Hoffmann PC, Bharat TAM, Wozny MR, Boulanger J, Miller EA, Kukulski W. Tricalbins contribute to cellular lipid flux and form curved ER-PM contacts that are bridged by rod-shaped structures. Dev Cell 2019;51(4):488-502.e8. pmid:31743663.

7. Kozminski KG, Alfaro G, Dighe S, Beh CT. Homologues of oxysterol-binding proteins affect Cdc42p- and Rho1p-mediated cell polarization in Saccharomyces cerevisiae. Traffic 2006;7(9):1224-42. pmid:17004323.

8. Audhya A, Emr SD. Regulation of PI4,5P2 synthesis by nuclear-cytoplasmic shuffling of the Mss4 lipid kinase. EMBO J 2003;22(16):4223-36. pmid:12912920.

9. Tavassoli S, Chao JT, Young BP, Cox RC, Prinz WA, de Kroon AI, Loewen CJ. Plasma membrane-endoplasmic reticulum contact sites regulate phosphatidylcholine synthesis. EMBO Rep 2013;14(5):434-40. pmid:23519169.

10. Gietz RD, Sugino A. New yeast-Escherichia coli shuttle vectors constructed with in vitro mutagenized yeast genes lacking six-base pair restriction sites. Gene 1988;74(2):527-34. pmid:3073106.
