# Supplementary figures and images for: ER-PM membrane contact site regulation by yeast ORPs and membrane stress pathways
Source: PLoS Genet. 2022 Mar 3;18(3):e1010106. doi: 10.1371/journal.pgen.1010106 (PMC8923467; doi:10.1371/journal.pgen.1010106)

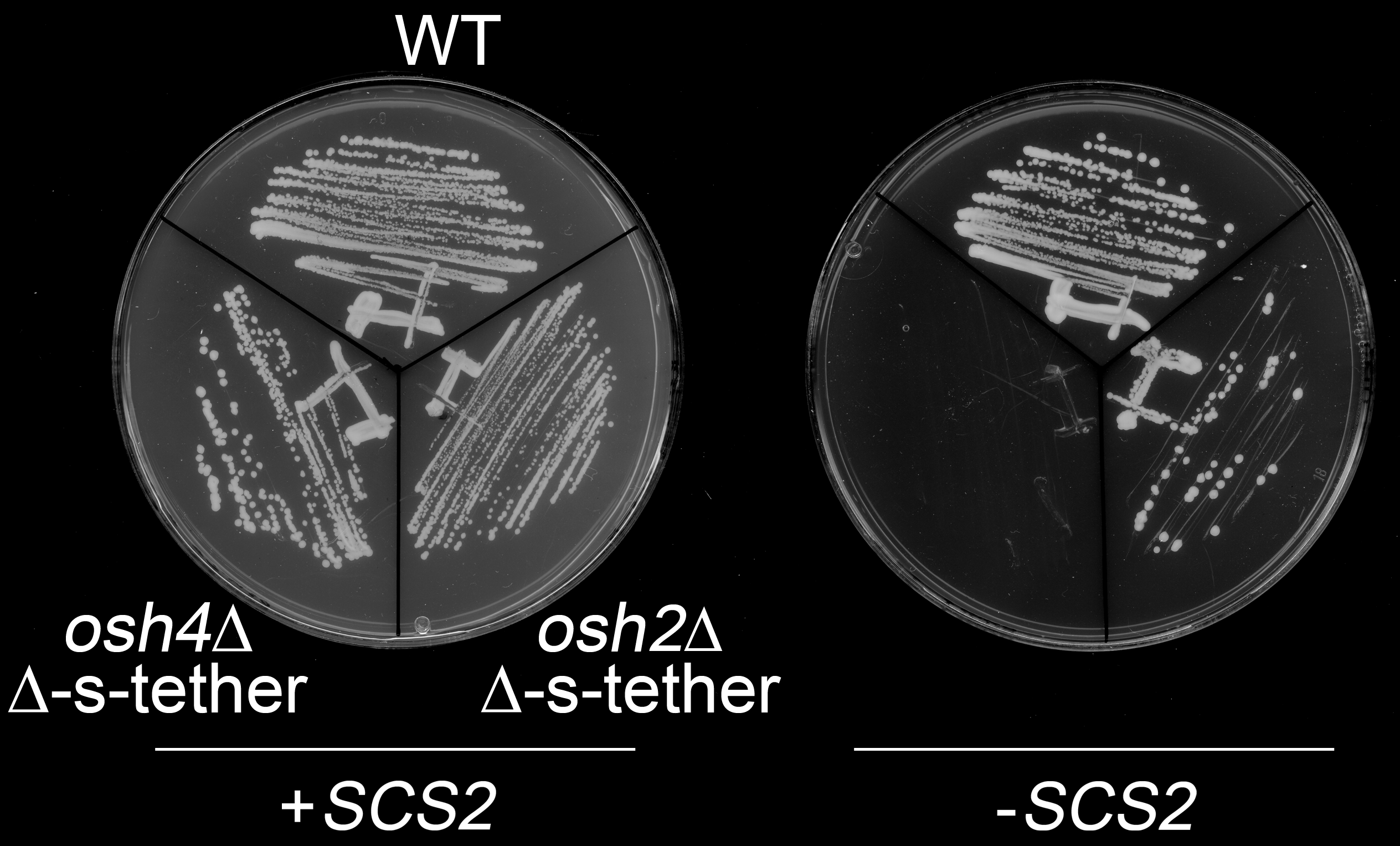

Supplement: S1 Fig — WT (SEY6210), osh4Δ Δ-s-tether (CBY5988) and osh2Δ Δ-s-tether (CBY6734) cells that contain SCS2 on a URA3-marked plasmid (pSCS2) were streaked onto solid growth medium. Cells were cultured for 4 days at 30°C on growth medium (containing 5’-FOA) to select against the SCS2-containing plasmid (-SCS2). As compared to growth on standard synthetic medium (+SCS2), osh4Δ Δ-s-tether cells do not growth in the absence of SCS2, whereas growth of osh2Δ Δ-s-tether cells is not dependent on SCS2. (TIF) [file pgen.1010106.s002.tif]

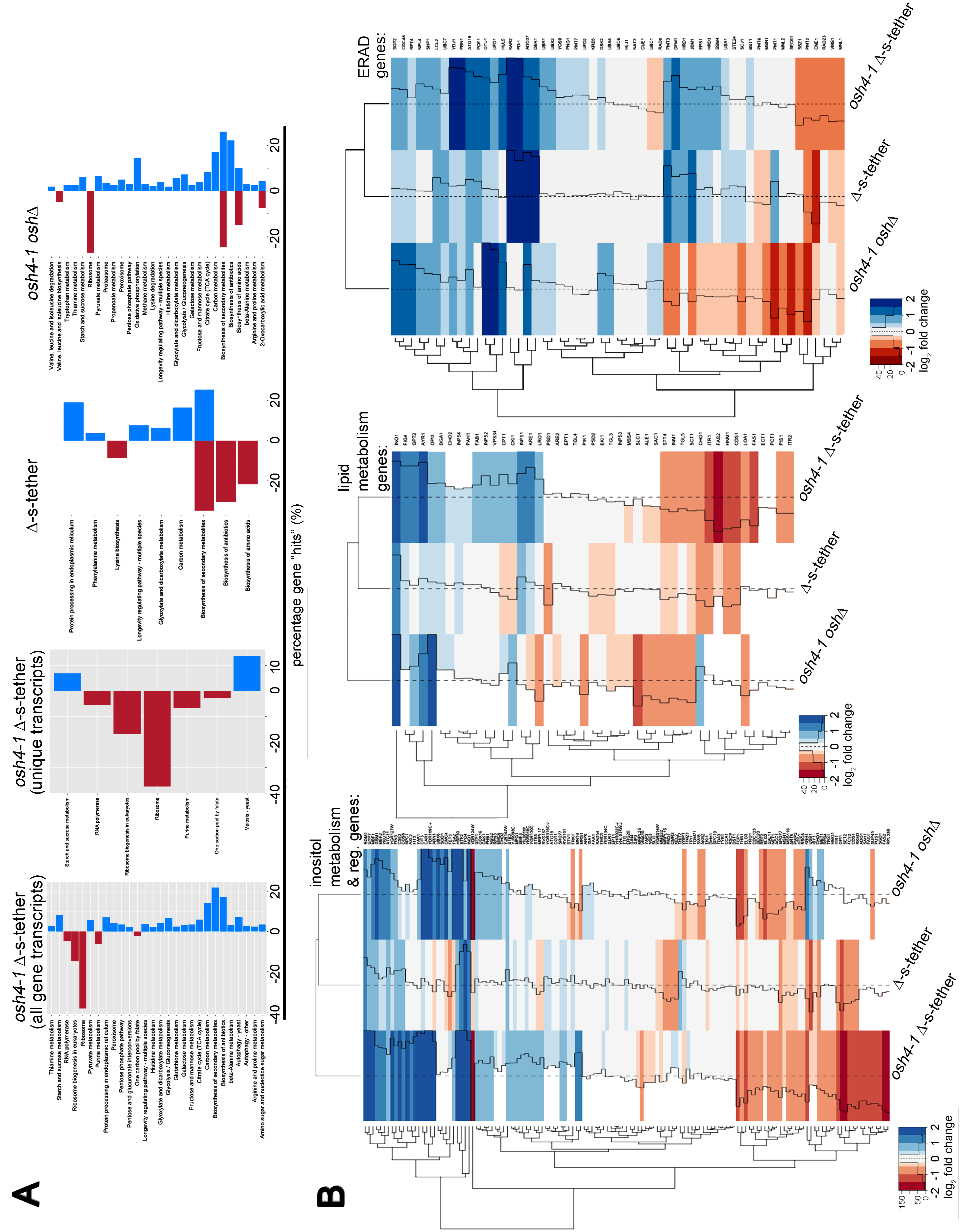

Supplement: S2 Fig — (A) Gene expression relative to WT (SEY6210) in osh4-1ts Δ-s-tether (CBY6031), Δ-s-tether (CBY5838), and osh4-1ts oshΔ (CBY926) cells listed by KEGG category. (B) Relative to WT, heatmap analyses of transcriptional responses in osh4-1ts Δ-s-tether, Δ-s-tether, and osh4-1ts oshΔ cells affecting inositol metabolism and regulation, lipid metabolism, and ERAD gene expression. Blue bars indicate transcriptional induction and red indicates repression. (TIFF) [file pgen.1010106.s003.tiff]

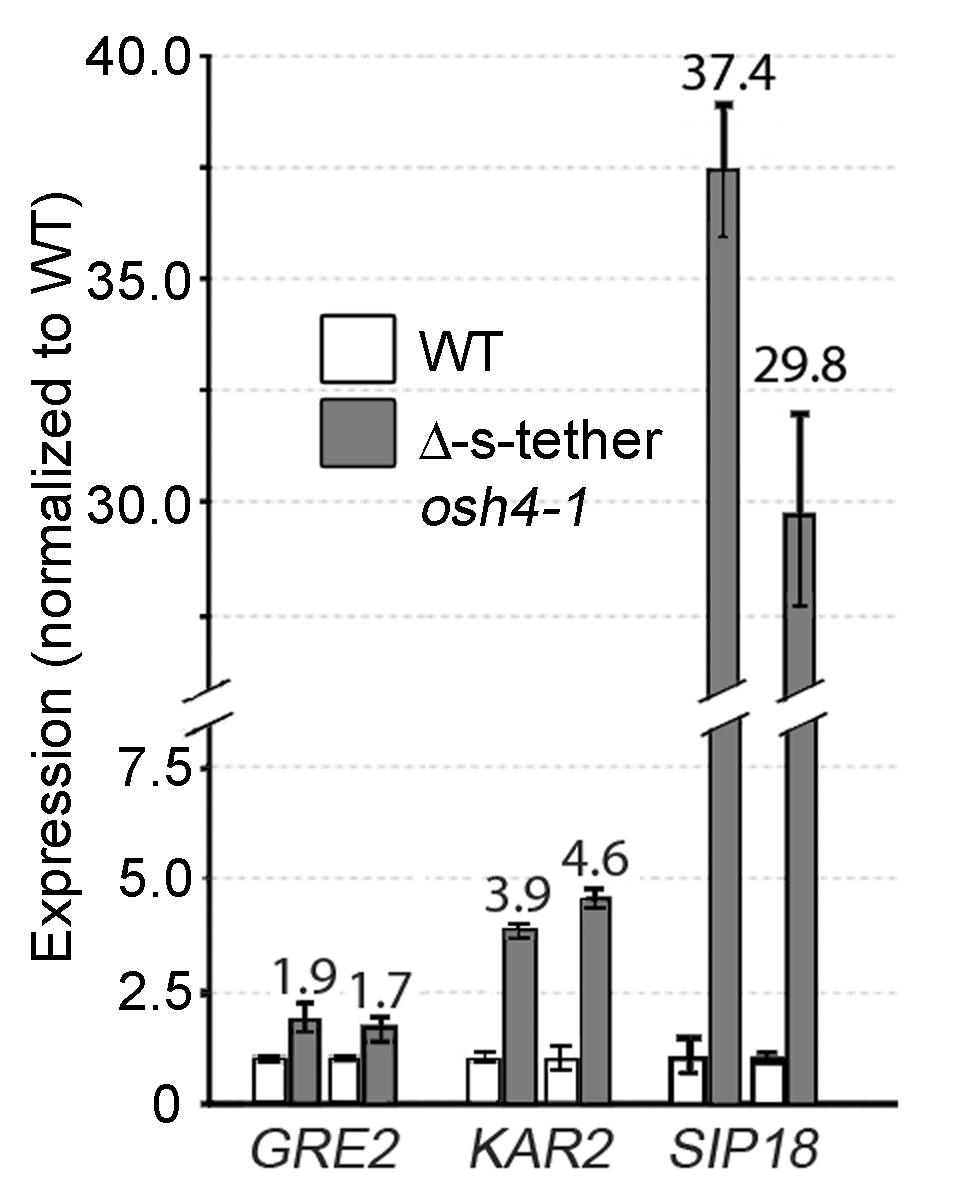

Supplement: S3 Fig — Duplicate analyses of GRE2, KAR2, and SIP18 transcript levels by RT-qPCR in WT (SEY6210) and osh4-1ts Δ-s-tether (CBY6031) cells at 37°C for 1 h. Each analysis represents three measurements, and error bars indicate SEM. (TIF) [file pgen.1010106.s004.tif]

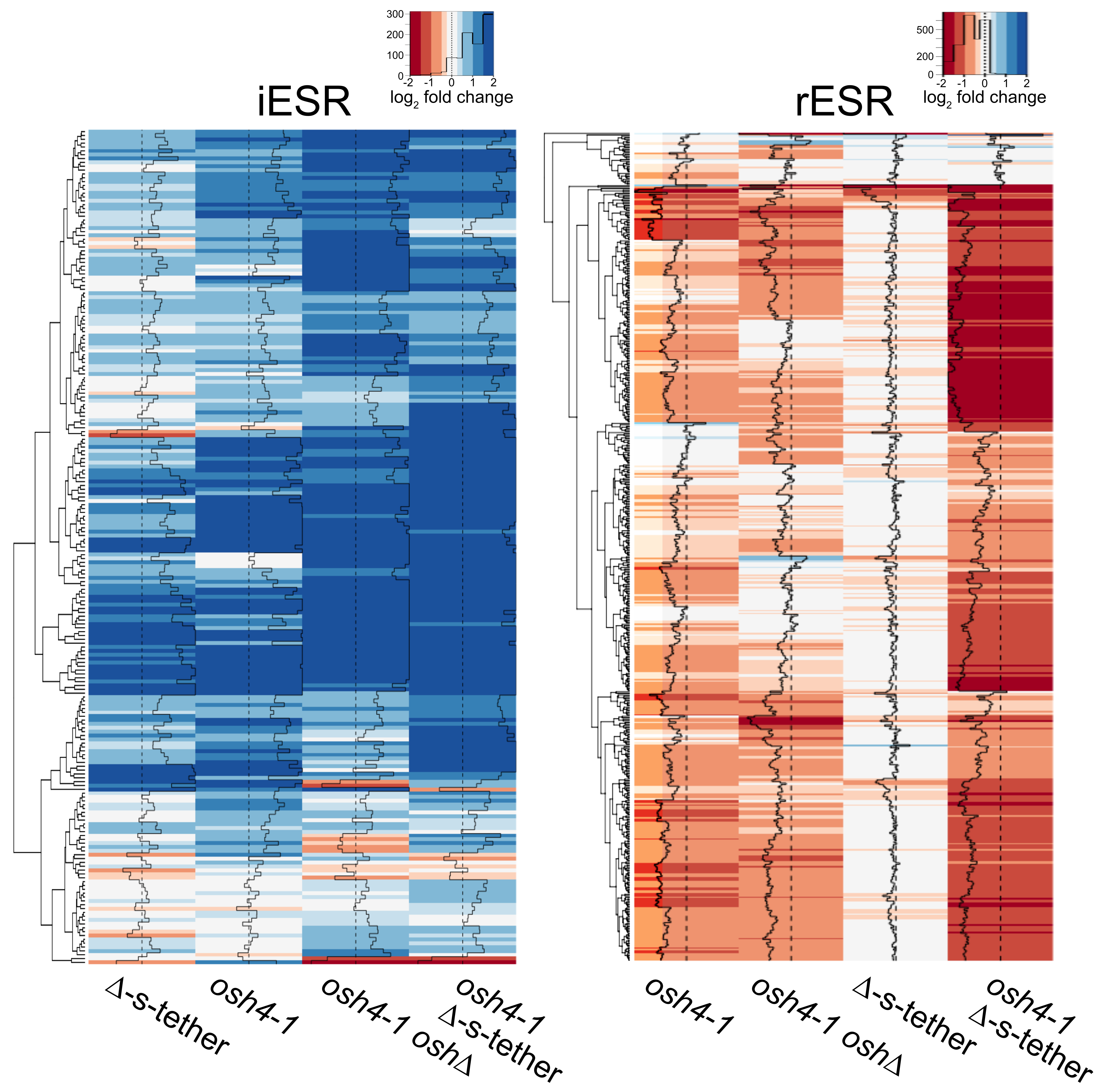

Supplement: S4 Fig — Heatmap analyses of transcriptional responses relative to WT (SEY6210) at 37°C for 1 h in osh4-1 (CBY7177), osh4-1ts Δ-s-tether (CBY6031), Δ-s-tether (CBY5898), and osh4-1ts oshΔ (CBY926) cells affecting (A) iESR and (B) rESR genes. Downregulated genes are shown in red, upregulated genes are shown in blue. iESR and rESR regulated genes were curated from previous reports [52,87]. (TIFF) [file pgen.1010106.s005.tiff]

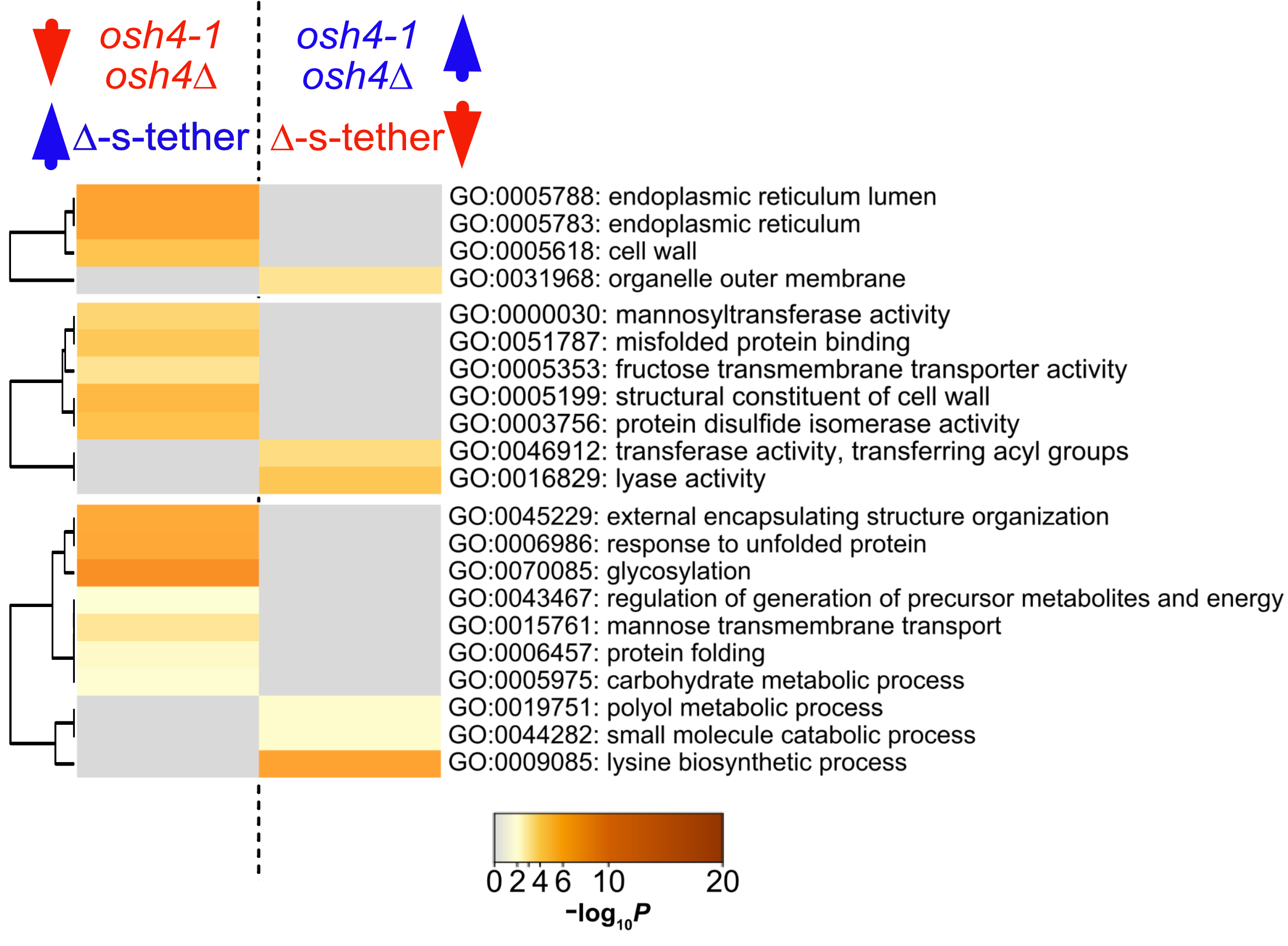

Supplement: S5 Fig — Anticorrelation of transcriptional responses in Δ-s-tether (CBY5898) versus osh4-1ts oshΔ (CBY926) cells. Reciprocal expression of specific GO-term gene groups are shown either when reduced in osh4-1ts oshΔ cells but increased in Δ-s-tether cells, or increased in osh4-1ts oshΔ cells and decreased in Δ-s-tether cells. (TIFF) [file pgen.1010106.s006.tiff]

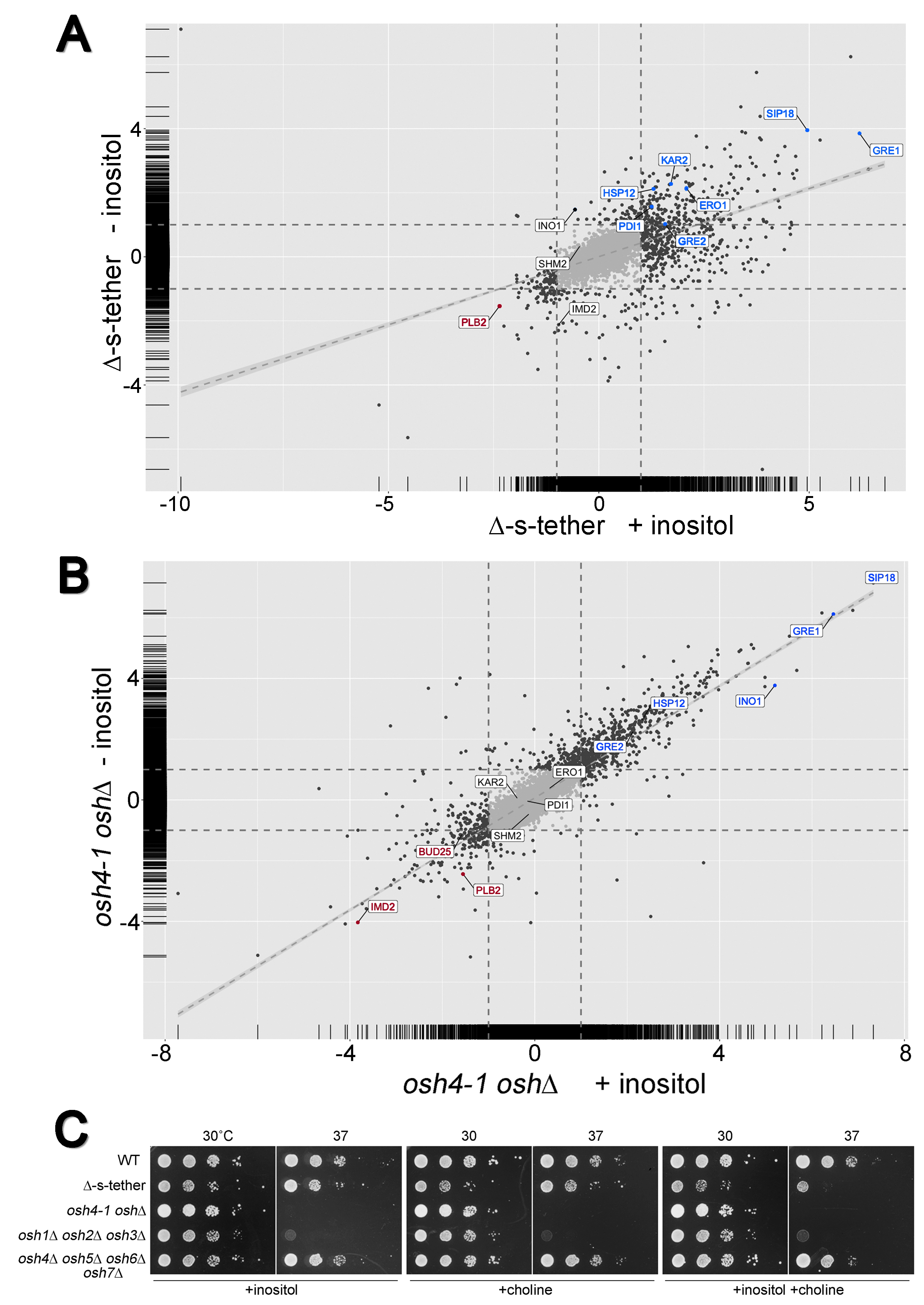

Supplement: S6 Fig — (A) Scatter plot analysis of gene expression in Δ-s-tether (CBY5838) cells, relative to WT (SEY6210), when cultured in the absence versus presence of exogenously added inositol. (B) Scatter plot analysis of osh4-1ts oshΔ (CBY926) gene expression, relative to WT, when cultured in the absence versus presence of exogenously added inositol. (C) Ten-fold serial dilutions of WT, Δ-s-tether, osh4-1ts oshΔ, osh1Δ osh2Δ osh3Δ (JRY6253), and osh4Δ osh5Δ osh6Δ osh7Δ (JRY6272) cells grown at 30 or 37°C on solid synthetic minimal media containing 75 μM myo-inositol, 1 mM choline, or 75 μM myo-inositol with 1 mM choline. (TIFF) [file pgen.1010106.s007.tiff]

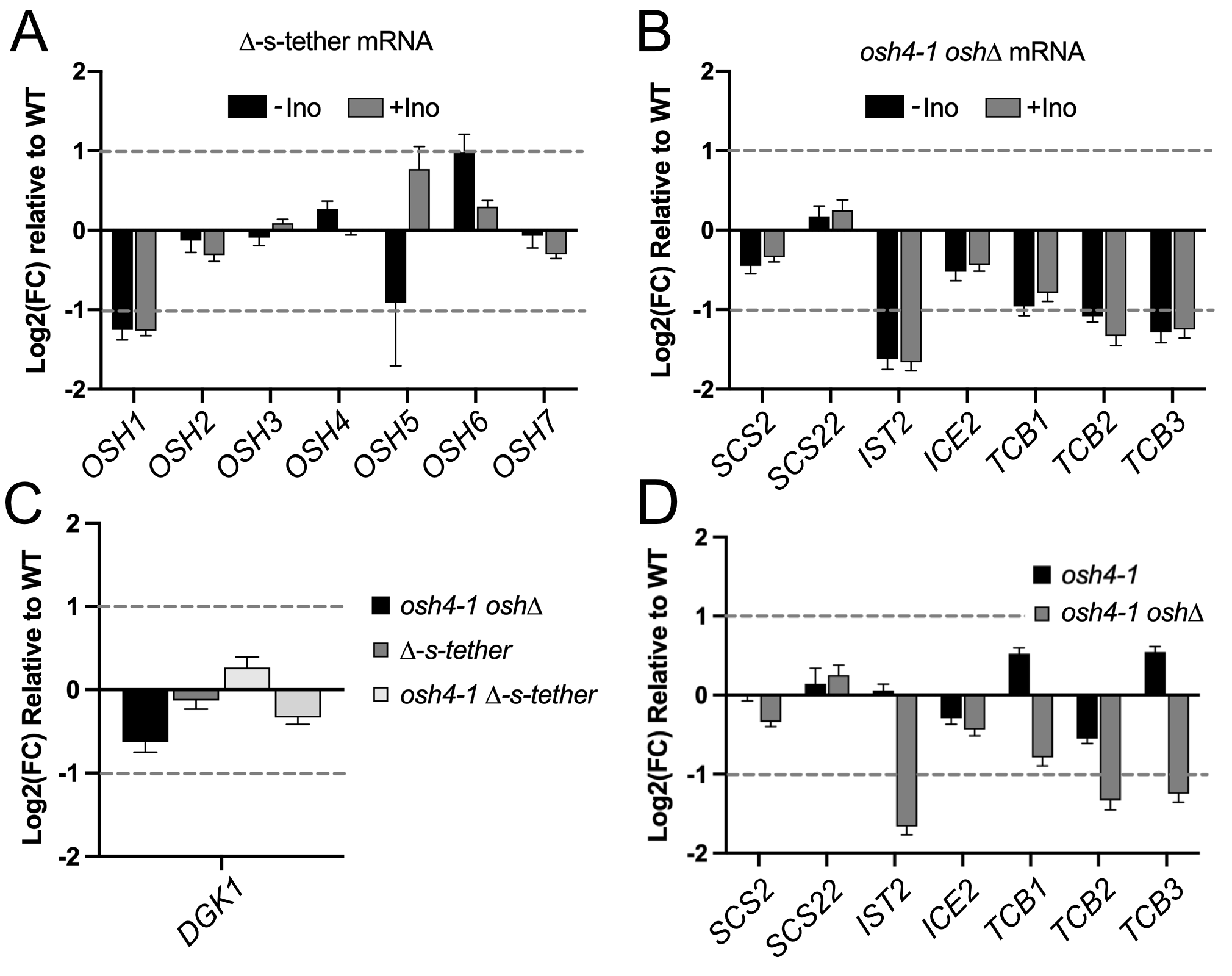

Supplement: S7 Fig — (A) Transcriptional expression of OSH1-OSH7 in Δ-s-tether (CBY5838) cells relative to WT (SEY6210) cells cultured with or without 75 μM inositol. (B) Transcriptional expression of genes encoding primary tether proteins in osh4-1ts oshΔ (CBY926) cells relative to WT cells cultured with or without inositol. (C) DGK1 expression in osh4-1ts oshΔ, Δ-s-tether, and osh4-1ts Δ-s-tether (CBY6031) cells relative to WT. (D) Transcriptional expression of ER-PM tether genes in osh4-1 (CBY7177) and osh4-1ts oshΔ cells relative to WT. (TIFF) [file pgen.1010106.s008.tiff]

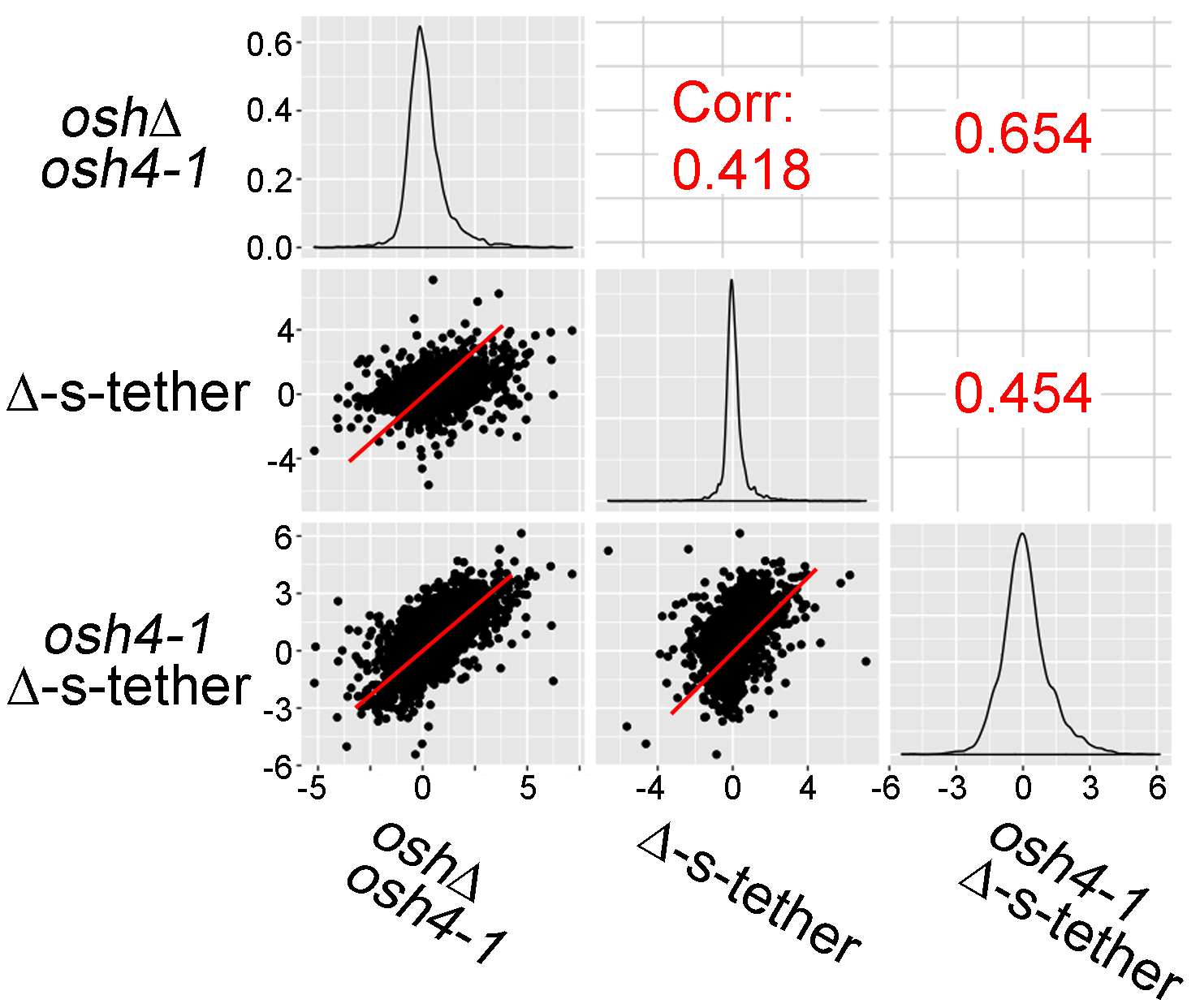

Supplement: S8 Fig — Correlation matrix of relative transcript abundance in osh4-1ts Δ-s-tether (CBY6031), Δ-s-tether (CBY5898), and osh4-1ts oshΔ (CBY926) cells relative to WT (SEY6210) grown in synthetic minimal medium at 30°C; osh4-1ts Δ-s-tether and osh4-1ts oshΔ cells were then incubated at 37°C for 1 h, as was their comparative WT control. Pearson correlations of corresponding genotypes as shown. (TIF) [file pgen.1010106.s009.tif]

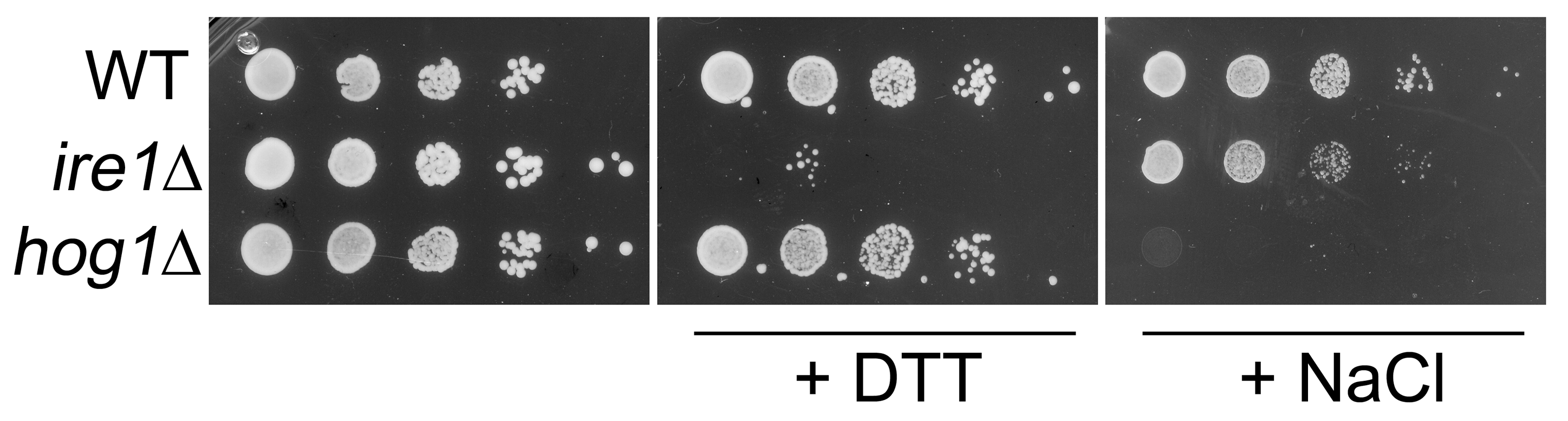

Supplement: S9 Fig — Ten-fold serial dilutions of WT, ire1Δ, and hog1Δ cells grown at 30°C on solid synthetic minimal media with and without 0.7 M NaCl or 4 mM DTT. Cells lacking IRE1 are sensitive to DTT but not to NaCl treatment and, inversely, cells lacking HOG1 are sensitive to NaCl treatment but not DTT treatment. (TIFF) [file pgen.1010106.s010.tiff]

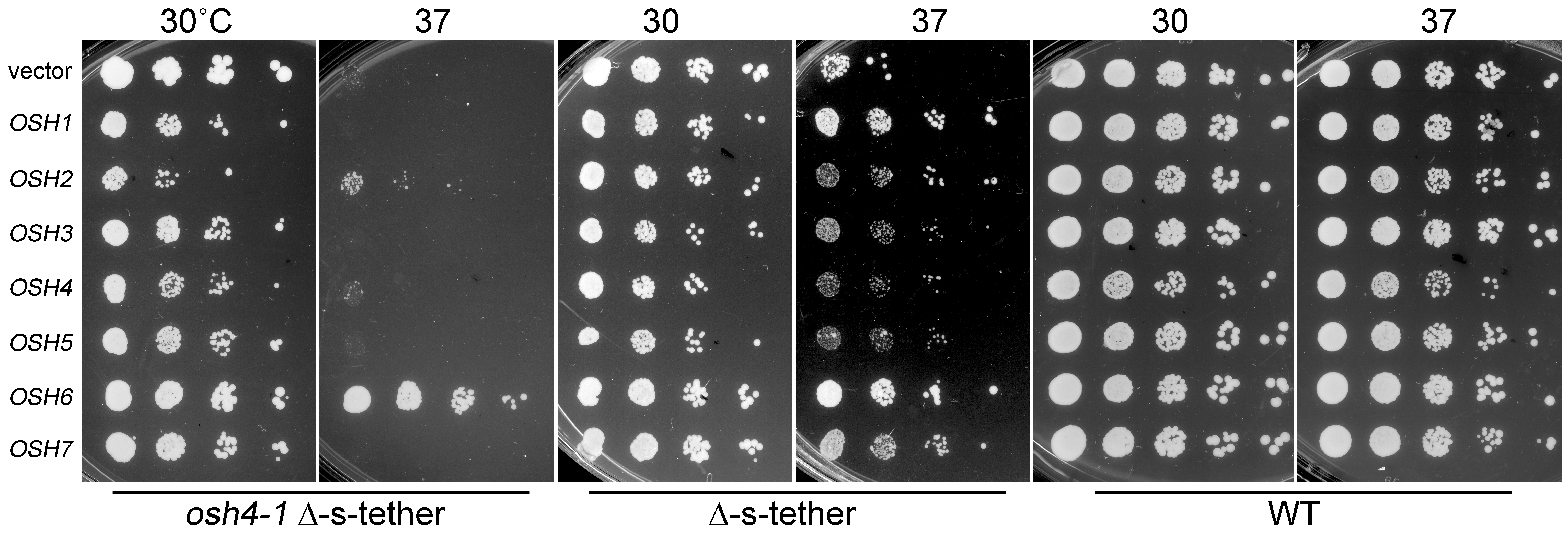

Supplement: S10 Fig — Tenfold serial dilutions of WT (SEY6210), Δ-s-tether (CBY5898), and osh4-1ts Δ-s-tether (CBY6031) transformed with the 2μ plasmid control (YEplac195), OSH1 (pCB240), OSH2 (pCB239), OSH3 (pCB238), OSH4 (pCB241), OSH5 (pCB242), OSH6 (pCB237), or OSH7 (pCB236). Cells were grown on synthetic complete medium for 3–5 days at 23 or 37°C (TIFF) [file pgen.1010106.s011.tiff]
